# Environmental Water and Sediment Microbial Communities Shape Intestine Microbiota for Host Health: The Central Dogma in an Anthropogenic Aquaculture Ecosystem

**DOI:** 10.3389/fmicb.2021.772149

**Published:** 2021-11-02

**Authors:** Zhijian Huang, Dongwei Hou, Renjun Zhou, Shenzheng Zeng, Chengguang Xing, Dongdong Wei, Xisha Deng, Lingfei Yu, Hao Wang, Zhixuan Deng, Shaoping Weng, Daliang Ning, Chuanle Xiao, Qingyun Yan, Jizhong Zhou, Zhili He, Jianguo He

**Affiliations:** ^1^State Key Laboratory of Biocontrol, Southern Marine Sciences and Engineering Guangdong Laboratory (Zhuhai), School of Marine Sciences, Sun Yat-sen University, Guangzhou, China; ^2^Institute of Aquatic Economic Animals and Guangdong Province Key Laboratory for Aquatic Economic Animals, School of Life Sciences, Sun Yat-sen University, Guangzhou, China; ^3^Department of Microbiology and Plant Biology, Institute for Environmental Genomics, School of Civil Engineering and Environmental Sciences, The University of Oklahoma, Norman, OK, United States; ^4^State Key Laboratory of Ophthalmology, Zhongshan Ophthalmic Center, Sun Yat-sen University, Guangzhou, China; ^5^Environmental Microbiomics Research Center, School of Environmental Science and Engineering, Sun Yat-sen University, Guangzhou, China

**Keywords:** microbial community, assembly mechanism, metacommunity, shrimp culture pond ecosystem, water/shrimp intestine/sediment habitat

## Abstract

From increasing evidence has emerged a tight link among the environment, intestine microbiota, and host health status; moreover, the microbial interaction in different habitats is crucial for ecosystems. However, how the environmental microbial community assembly governs the intestinal microbiota and microbial communities of multiple habitats contribute to the metacommunity remain elusive. Here, we designed two delicate experiments from temporal and spatial scales in a shrimp culture pond ecosystem (SCPE). Of the SCPE metacommunity, the microbial diversity was mainly contributed to by the diversity of^–^β_*IntraHabitats*_ and β_*InterHabitats*_, and water and sediment communities had a large contribution to the shrimp intestine community as shown by SourceTracker and Sloan neutral community model analyses. Also, phylogenetic bin-based null model results show that microbial assembly of three habitats in the SCPE appeared to be largely driven by stochastic processes. These results enrich our understanding of the environment–intestinal microbiota–host health closely linked relationship, making it possible to be the central dogma for an anthropogenic aquaculture ecosystem. Our findings enhance the mechanistic understanding of microbial assembly in the SCPE for further analyzing metacommunities, which has important implications for microbial ecology and animal health.

## Introduction

The intestinal microbiome is increasingly recognized as having a fundamental role in regulating the physiology and health of animals and humans (Clemente et al., 2012; Le Chatelier et al., 2013; Jie et al., 2017). Generally, both host genetics and the environment can shape the composition of the human intestinal microbiota (Benson et al., 2010; Spor et al., 2011; Turpin et al., 2016), and moreover, a recent study demonstrates that the effect of environmental factors appears to outweigh host genetics in shaping the microbiota (Rothschild et al., 2018). Thus, a central issue is the extent to which the intestinal microbial community is determined by host genetics and/or the environment. Increasing evidence has emerged that the composition of the intestine microbiome is shaped by multiple factors and supports a close link among the environment, intestinal microbiota, and host health status (Rothschild et al., 2018; Sun et al., 2020), which is similar to the central dogma of molecular biology (Crick, 1970). It has been long appreciated that microorganisms play an indispensable role in various ecosystems and provide a wide range of environmental services (Wall et al., 2015; Graham et al., 2016; Fierer, 2017), including maintaining the quality of aquatic ecosystems, as this has an impact on animal health and disease control in addition to element cycling and water quality, and affects the productivity and sustainability of aquaculture (Moriarty, 1997; Assefa and Abunna, 2008; Zhou et al., 2009; Tania et al., 2018). More importantly, microbial communities from multiple habitats in aquaculture systems (e.g., surrounding water, animal intestine, and sediment) are all closely related to the occurrence of aquatic animal diseases (Zhu et al., 2016; Hou et al., 2017, 2018; Xiong et al., 2017), challenging us to fully understand the structure, function, and interaction in such complex aquaculture ecosystems.

With increasing demands for animal proteins due to rising populations, global aquaculture production has increased by 500% since the late 1980s (Food and Agriculture Organization, 2016). Aquaculture has become the third largest source of animal proteins, accounting for 17% of global protein consumption, and the annual output of aquaculture products in China is more than 60% of the world’s (Food and Agriculture Organization, 2018). Unfortunately, the frequent occurrence of diseases has threatened the aquaculture industry. Shrimp (mainly *Litopenaeus vannamei* and others) are among the most important aquatic products among fishery trading commodities worldwide (Zhang et al., 2019). Recently, bacterial diseases, such as white feces syndrome, early mortality syndrome, acute hepatopancreatic necrosis disease, and hepatopancreas necrosis syndrome, have reduced the global production of shrimp by an estimated 23%, leading to a loss of billions of dollars annually (Sriurairatana et al., 2014; Lee et al., 2015; Huang et al., 2016, 2020). The contribution of dysbiosis in intestinal microbiota to human and animal diseases is recognized (Jie et al., 2017; Zhang et al., 2018; Huang et al., 2020). As a unique anthropogenic aquaculture ecosystem, the shrimp culture pond ecosystem (SCPE) is disturbed by artificial manipulation and management and is composed of many biotic and abiotic factors in multiple habitats (e.g., water, shrimp intestine, sediment, and so on), especially with aquatic animals living in the ecosystem, forming a metacommunity, which is different from other natural and engineered ecosystems (Huang et al., 2016; Hou et al., 2018). Therefore, it is necessary to understand the microbial ecology of the SCPE metacommunity for sustainable outputs of aquaculture products.

Microbial communities in aquaculture ecosystems, as in many types of habitats (Rungrassamee et al., 2014; Fan et al., 2016; Hou et al., 2018), are highly diverse and vary concurrently with various environmental and geographic factors (e.g., host developmental stages, environmental factors, and geographical distance) (Yan et al., 2016; Hou et al., 2017; Li et al., 2017; Zeng et al., 2017). Despite recent advances in understanding the microbial ecology of aquaculture ecosystems, their microbial assembly mechanisms remain unclear. In general, the mechanisms shaping the microbial diversity among species are considered to be ecological processes (Hanson et al., 2012). Recently, our knowledge about ecological processes in shaping the microbial community has been enriched substantially (Zhou and Ning, 2017; Ning et al., 2019, 2020). For some aquatic ecosystems (e.g., lakes), deterministic processes play a primary role in shaping the water or sediment microbial community structure (Wang et al., 2013; Yan et al., 2017). Stochastic processes play a dominant role in the assemblage of microbial communities in aquatic animal intestines (Burns et al., 2016). More importantly, in aquatic ecosystems, microbial communities of water, animal intestine, sediment habitats, and other associated habitats constitute a metacommunity (Al-Harbi and Uddin, 2005; Leibold et al., 2014; Del’Duca et al., 2015; Cleary et al., 2019), and although still poorly understood, the interaction among these communities is important for aquatic animal productivity and health (Schryver and Vadstein, 2014). In particular, it is essential to understand the interaction among microbial communities of animal intestine and surrounding environments for healthy aquaculture. However, how microbial communities of multiple habitats (water, animal intestine, and sediment) in aquaculture ecosystems contribute to the metacommunity and the ecological interplay between environmental communities and intestinal microbiota is poorly understood.

In this study, we aimed to understand microbial assembly mechanisms for a metacommunity of three habitats (water, shrimp intestine, and sediment) in SCPE with three ecological questions: (i) What ecological processes shape the microbial community structures in the three habitats? (ii) What is the contribution of communities from each habitat to the SCPE metacommunity? (iii) Is the shrimp intestinal microbiota shaped by environmental microbial communities? To address these questions, we hypothesized that (H1) the communities of three habitats have important contributions to the SCPE microbial metacommunity and (H2) environmental microbial communities have a decisive role in shaping the shrimp intestinal microbiota.

To address these hypotheses, we analyzed microbial communities from the three habitats (water, shrimp intestine, and sediment) in SCPE across six regions in China, tracked the dynamics of microbial communities of six development stages in the entire cycle of shrimp culture, and explored their assembly mechanisms by sequencing of 16S rRNA gene amplicons and metacommunity analysis. This study provides new insights into our understanding of microbial assembly mechanisms in the SCPE, and the developed framework will facilitate metacommunity analysis, significantly advancing microbial ecology of aquaculture ecosystems and animal health.

## Materials and Methods

### Experimental Design and Sample Collection in Shrimp Culture Pond Ecosystem

Samples were collected from 88 *L. vannamei* cultural ponds in six regions, i.e., Dianbai and Yangjiang (DB + YJ), Zhuhai and Zhongshan (ZH + ZS), Hainan (HN), Qinzhou (QZ), Zhangpu (ZP), and Tianjing (TJ) (19.20°–39.29°N, 108.56°–117.93°E) in China over the period of June to October, 2017. Geographical distances between sites ranged from 0.31 to 2063.35 km. The sampled ponds had similar size (∼3300 m^2^), water depth (∼1.2 m), shrimp development stage (50–60 days), and stocking density (100,000–200,000 shrimps each pond) (Supplementary Table 1). Site locations were recorded by global positioning system (GPS) (Garmin Vista HCx, United States).

Each water sample (0.5 L) was taken from a depth of 0.5 m below the surface using a sterile bottle, and samples were immediately placed on ice before filtration through a 0.22-μm polyethersulfone membrane (Supor-200, Pall Corporation, Washington, NY, United States) using a vacuum pump (Hou et al., 2017). The surface of shrimp was sterilized with 70% ethanol, and then intact intestine was aseptically dissected from the musculature and placed into a 15-mL sterile centrifuge tube containing 10 mL PBS buffer (Zeng et al., 2017). Each 1.0-g sediment sample was placed into a 15-mL centrifuge tube and washed with 10 mL PBS buffer three times. Three water/shrimp intestine/sediment samples were collected in each pond. All samples were stored at −80°C until DNA extraction (Hou et al., 2018).

Water temperature, pH, dissolved oxygen (DO), and salinity were measured on-site using a YSI handheld multiparameter instrument (Model YSI 380, YSI Incorporated, United States). Sediment pH was measured on-site using a soil pH meter (ZD-05, Beijing Century Euron Co., Ltd., China). The total nitrogen (TN), total phosphorus (TP), dissolved inorganic nitrogen [ammonia nitrogen (NH_4_^+^-N), nitrite nitrogen (NO_2_^–^-N) and nitrate nitrogen (NO_3_^–^-N)], and orthophosphate (PO_4_^3–^-P) of water samples and TN and TP of sediment samples were measured using an auto discrete analyzer (Model CleverChem 380, DeChem-Tech, Germany). The total carbon (TC) and total organic carbon (TOC) of water and sediment samples were measured using a TOC analyzer (Aurora 1030W, OI Analytical, United States). All physicochemical variables of water and sediment samples are described in Supplementary Tables 2, 3.

To further verify the contribution of microbial communities of multiple habitats to the SCPE metacommunity, we conducted the experiment to explore the temporal dynamics of environmental water and sediment microbiota and intestinal microbiota along *L. vannamei* development. For the delicate experiment, we selected nine shrimp culture ponds in the study area, which was located at Lianxi shrimp farm of Guangdong Haida Group Co., in Zhuhai, China (22.37°N, 113.22°E). Each pond had similar size (∼3300 m^2^), water depth (∼1.2 m), and stocking density (∼100,000 shrimp) with strict and uniform culture management. Sampling was carried out at 0, 10, 20, 30, 40, and 50 days post-larval shrimp inoculation (DPI) (named day 0, day 10, day 20, day 30, day 40, and day 50 groups, respectively) during shrimp culture development stage (Supplementary Table 4). Water, shrimp intestine, and sediment samples of each pond were processed and collected accordingly in the same way as those in the six areas mentioned above. The physicochemical factors were also measured with analyzers (Supplementary Tables 5, 6). In total, 162 samples were collected and stored at −80°C prior to DNA extraction.

### DNA Extraction, Polymerase Chain Reaction (PCR) Amplification, and 16S rRNA Gene Amplicon Sequencing

Genomic DNA from water, shrimp intestine, and sediment samples were extracted using the Water DNA Isolation Kit (Omega Bio-Tek, Doraville, GA, United States), PowerFecal DNA Isolation Kit (Mobio, Carlsbad, CA, United States), and PowerSoil DNA Isolation Kit (MO BIO, Carlsbad, CA, United States), respectively. The 338F and 806R (5′-ACTCCTACGGGAGGCA GCAG-3′ and 5′-GGACTACHVGGGTWTCTAAT-3′) universal primer pair was used to amplify the V3–V4 regions of the bacterial 16S rRNA gene. The PCR products from the samples were equally combined and then sequenced using the Illumina MiSeq platform (Illumina, San Diego, CA, United States) by Majorbio Bio-Pharm Technology Co., Ltd. (Shanghai, China). Raw sequencing data were deposited in the NCBI Short Read Archive, BioProjectID PRJNA545396 and PRJNA689351.

Paired-end sequences were merged using FLASH (V1.2.11) (Magočm and Salzberg, 2011), and merged sequences were processed following the Quantitative Insights Into Microbial Ecology pipeline (QIIME, version 1.9.0) (Caporaso et al., 2010). In brief, the sequences with ambiguous bases or truncated at any site of more than three consecutive bases receiving a Phred quality score (Q) <20 were removed. Chimeric sequences were discarded using the UCHIME algorithm (Edgar et al., 2011). Sequences with a distance-based identity of 97% or greater were grouped into operational taxonomic units (OTUs) using UCLUST (Edgar, 2010). The most abundant sequence from each OTU was selected as representative and then was taxonomically assigned against the Silva SSU database 128 using the RDP Classifier algorithm, which enables each identified OTU to have a close relative. To correct for uneven sequencing efforts, the OTU table for bacteria was 10× randomly rarefied to a subset of 14,435 sequences per sample in subsequent analyses. The core OTU was defined based on multiple reported measures: OTU with an occurrence frequency in more than 90% of all samples (Ugland and Gray, 1982; Ainsworth et al., 2015). Following the same criteria as described above, core OTUs in each habitat were identified from water, shrimp intestine, and sediment samples in the SCPE.

### Relationships Among Water, Shrimp Intestine, and Sediment Microbial Communities in the Shrimp Culture Pond Ecosystem

The relationship between microbial communities (habitat, location, development stage) was analyzed using Venn analysis based on the detected OTUs (Edwards et al., 2014). For location and development stage, an additive partitioning framework was applied to separate out the total microbial diversity at the ecosystem level (Ɣ_*Ecosystem*_) into smaller scale contributions from habitats to local communities (Escalas et al., 2013). More precisely, total ecosystem microbial diversity was expressed as the sum of the inter-habitat difference in the community diversity, the mean intra-habitat difference, and mean local community diversity with Ɣ_*Ecosystem*_ = β_*InterHabitats*_ + $\bar{\beta }$_*IntraHabitats*_ + $\bar{\alpha }$_*LocalCommunities*_. The ecosystem level (Ɣ_*Ecosystem*_) may arise from a high microbial dissimilarity among ponds (β_*InterHabitats*_), a high dissimilarity among communities within each pond ($\bar{\beta }$_*IntraHabitats*_), or from a high diversity within each local community ($\bar{\alpha }$_*LocalCommunities*_; i.e., each water, shrimp intestine, and sediment sample). To further evaluate the relationships among microbial communities, the different sources were used to estimate their contributions to microbial community composition of the SCPE using SourceTracker based on a Bayesian algorithm (Knights et al., 2011), which was run through QIIME with default settings and with one habitat as the sink and the other two habitats as sources. The Sloan neutral community model (Sloan et al., 2006) was used to analyze the OTUs that were shared between the shrimp intestine and surrounding water and/or sediment, in which the microbial community in water and sediment was the source of intestinal microbiota. This model predicts that the probability of detecting an OTU in shrimp intestine due to dispersal is directly proportional to its abundance in the corresponding water and/or sediment community. OTUs were sorted into three categories depending on whether they occur more frequently (overrepresented), less frequently (underrepresented), or within (neutrally distributed) the 95% confidence interval of the neutral model predictions.

### Estimation of Ecological Processes and Microbial Ecological Succession in Shrimp Culture Pond Ecosystem

We used the inferred community assembly mechanisms by a phylogenetic bin-based null model (iCAMP) (Ning et al., 2020) to evaluate the contribution of ecological processes on microbial assembly of the three habitats in the SCPE based on location and development stage. First, the observed taxa were divided into 24 “bins” based on their phylogenetic relationships. Then, the process governing each bin was identified based on null model analysis of phylogenetic diversity using a beta net relatedness index (βNRI) and taxonomic β-diversities using modified Raup-Crick metric (RC). For each bin, the fraction of pairwise comparisons with βNRI <−1.96 and >+1.96 were considered as the percentages of homogeneous and heterogeneous selection, respectively. Next, RC is used to partition the remaining pairwise comparisons with | βNRI| ≤ 1.96: The fraction of pairwise comparisons with RC <−0.95 and >+0.95 are treated as the percentages of homogenizing dispersal and dispersal limitation, and remains with | βNRI| ≤ 1.96 and | RC| ≤ 0.95 represent the percentages of drift. The above analysis was repeated for every bin, and then the fractions of individual processes across all bins were further weighted by the relative abundance of each bin and summarized to estimate the relative importance of individual processes at the whole community level.

### Statistical Analysis

A ternary plot was applied to reveal the distribution of the dominant genera (>0.1%) among water, shrimp intestine, and sediment habitats using the package “ggtern” in R 3.3.2 (R Core Team, 2015). Welch’s *t*-test was used to compare the microbial diversity indices among water, shrimp intestine, and sediment habitats by location and development stage. The non-metric multidimensional scaling (NMDS) and analysis of similarity (ANOSIM) were performed to evaluate the overall differences in microbial communities of water, shrimp intestine, and sediment habitats using the Bray–Curtis distance (Li et al., 2017). Then, the differentially abundant taxa among three habitats were identified using one-way analysis of variance (one-way ANOVA) (Cleary et al., 2019). Moreover, we employed molecular ecology network analysis (Deng et al., 2012) to evaluate the extent of microbial interspecies interactions of water, shrimp intestine, and sediment habitats, respectively, across six regions or six culture development stages. To quantify the interspecies interactions, a set of topological properties were calculated, including the average path length, clustering coefficient, and co-occurrences (Mej, 2003), and the resulting network was visualized via Cytoscape 3.6.1.^1^ The structure equation model (SEM) analysis (Bagozzi and Yi, 2012) was used to illustrate the interplay rearing water sediment and shrimp intestinal microbial communities and implement the effect of water and sediment environmental factors on their microbial communities.

## Results

### Microbial Community Diversity of the Three Habitats in the Shrimp Culture Pond Ecosystem Across the Country

**FIGURE 1:**
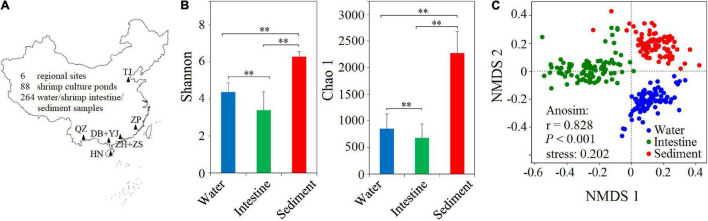
Sampling sites and the α- and β-diversity of microbial communities from three habitats. **(A)** A total of 264 water, shrimp intestine, and sediment samples (88 samples in each habitat) were collected from 88 cultural ponds in six regions in China. DB + YJ, ZH + ZS, HN, QZ, ZP, and TJ indicate Dianbai + Yangjiang, Zhuhai + Zhongshan, Hainan, Qingzhou, Zhangpu, and Tianjin. **(B)** The α-diversity of microbial communities among water, shrimp intestine, and sediment habitats in the SCPE of six regions. Statistical significance of the α-diversity indices among three habitats were based on the Welch’s *t*-test (^∗∗^: *P* < 0.01). **(C)** The β-diversity of microbial communities of three habitats analyzed by NMDS and ANOSIM based on the Bray–Curtis distance.

To understand the microbial diversity in the SCPE, we conducted a large-scale sampling and took water, shrimp intestine, and sediment samples in 88 shrimp cultural ponds in six regions (Figure 1A). We extracted DNA from all 264 samples and sequenced their 16S rRNA gene amplicons. A total of 3,810,840 high-quality sequences were obtained from all samples. The sequences clustered into 7656 OTUs with the highest number (i.e., 7389) in the sediment (Supplementary Table 7). This was enough to capture a majority of the microbial communities in all samples with a coverage index of 0.96–0.99 (Supplementary Table 7). The Shannon index significantly (*P* < 0.001) differed among those three habitats with the highest in the sediment (6.28 ± 0.28), followed by water (4.37 ± 0.50) and then shrimp intestine (3.39 ± 0.99) habitats. The Chao1 index showed similar results (Figure 1B and Supplementary Table 7). To further evaluate the overall differences among three habitats, NMDS analysis showed that microbial communities clustered based on habitat, and ANOSIM analysis further revealed that the microbial structures differed significantly (*r* = 0.828, *P* < 0.001) between any two of the habitats (Figure 1C).

### Core Microbial Operational Taxonomic Units in Each Habitat of the Shrimp Culture Pond Ecosystem at Six Regions Across the Country

We next examined the occurrence of taxa in each SCPE to determine if the core microbial taxa existed in each habitat. We defined the core taxa as those taxa that occurred in ≥90% of water, shrimp intestine, and sediment samples, respectively. The results showed that about 0.6% (28 of 5078 OTUs), 0.6% (23 of 3919 OTUs), and 0.4% (30 of 7389 OTUs) of the OTUs constituted core taxa in the water, shrimp intestine, and sediment habitats, respectively. This accounted for 33.1, 48.1, and 7.7% of all sequences obtained (Supplementary Table 8). The core OTUs in the water belonged to the phyla Cyanobacteria (19.7%), Actinobacteria (7.4%), Proteobacteria (1.2%), Verrucomicrobia (0.8%), and Bacteroidetes (4.1%); in the shrimp intestines to Proteobacteria (36.6%), Cyanobacteria (3.6%), Actinobacteria (0.4%), Tenericutes (7.0%), and Verrucomicrobia (0.5%); and in the sediments to Proteobacteria (2.9%), Bacteroidetes (3.2%), Actinobacteria (0.8%), Cyanobacteria (0.6%), Chloroflexi (0.2%), and Deinococcus Thermus (0.1%) (Supplementary Table 8). Although there were some overlaps of phyla in the different core communities, overall, the core communities from each habitat were distinct, suggesting that each habitat would select their core taxa. Twenty-three core OTUs from the shrimp intestine were also present in the water and/or sediment habitats (Supplementary Table 8), suggesting possible sources (e.g., environmental water and sediment) of shrimp intestinal microbial communities. Additionally, several known opportunistic pathogens in aquatic ecosystems, *Photobacterium* OTU3557, *Vibrio* OTU1384, *Vibrio* OTU1482, *Vibrio* OTU2357, *Vibrio* OTU2482, and *Candidatus* Bacilloplasma OTU1192, were members of the shrimp intestine core community (Supplementary Table 8).

### Comparison of the Microbial Composition of Three Habitats in the Shrimp Culture Pond Ecosystem in Six Regions

**FIGURE 2:**
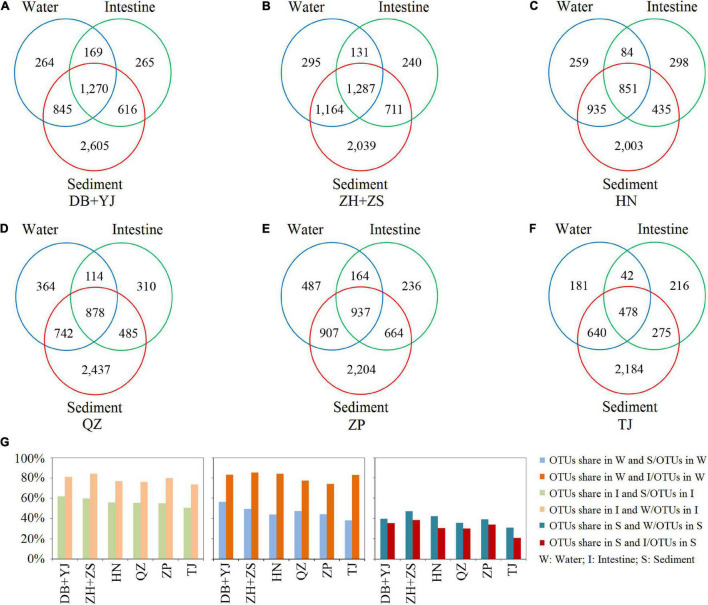
Venn analysis of microbial composition in water, shrimp intestine, and sediment habitats based on detected OTUs of six regions. **(A–F)** The numbers of OTUs in each habitat and shared in any two or three habitats. **(G)** The percentage of OTUs shared in any two habitats.

To understand the microbial composition of the water, sediment, and shrimp intestine in SCPEs in six regions, we compared the OTUs present in each using Venn analysis. The results show that many OTUs were commonly present in all three habitats, and the number of OTUs was found to be in any two habitats of each regional site (Figure 2). For example, at the DB + YJ site, 1270 OTUs were present in all three habitats, and some OTUs were found to be in any two habitats: 1439 (intestine, 56.5%) or 3015 (sediment, 83.0%) out of 2548 water OTUs; 1439 (water, 62.0%) or 1886 (sediment, 81.2%) out of 2320 intestinal OTUs; 1886 (intestine, 35.3%) or 3015 (water, 39.6%) out of 5336 sediment OTUs (Figures 2A,G). Most OTUs detected at each site were present in the sediment habitat, and a high percentage (∼80%) of those were also present in the shrimp intestine and water habitats. Similar trends were observed in each pond (Supplementary Figure 1).

Further comparison showed that most of the detected phyla and genera were always present in at least one of the three habitats but that their relative abundances significantly (*P* < 0.001) differed (Supplementary Figures 2, 3Supplementary Tables 9–11). Specifically, some opportunistic pathogens, such as *Vibrio*, *Photobacterium*, and *Candidatus* Bacilloplasma, were detected in all the three habitats, but the relative abundance in the shrimp intestine was significantly (*P* < 0.001) higher than in the other two habitats (Supplementary Figure 3). Additionally, we compared microbial co-association networks of the three habitats. The average degree indices of the water, shrimp intestine, and sediment microbial communities were 14.55, 14.94, and 11.25, and the average clustering coefficient index values were 0.57, 0.51, and 0.71 with an average path distance of 1.87, 1.89, and 2.02, respectively (Supplementary Figure 4 and Supplementary Table 12). These results reveal that the microbial network in shrimp intestine is more complex and better connected than in the water and sediment habitats.

We were able to identify several keystone species in these habitats. Keystone species were those with the largest number of connections. OTU4327 and OTU16092, each with 37 connections, were classified as the keystone species with the highest degree nodes and numerous neighbors in the water habitat; OTU9882 in the shrimp intestine had 50 connections; and OTU16554 in the sediment had 79 connections (Supplementary Figure 4 and Supplementary Table 13).

### Microbial Communities of Environmental Water and Sediment Mainly Contribute to Shrimp Intestinal Microbiota in the Shrimp Culture Pond Ecosystem Metacommunity in Six Regions

**FIGURE 3:**
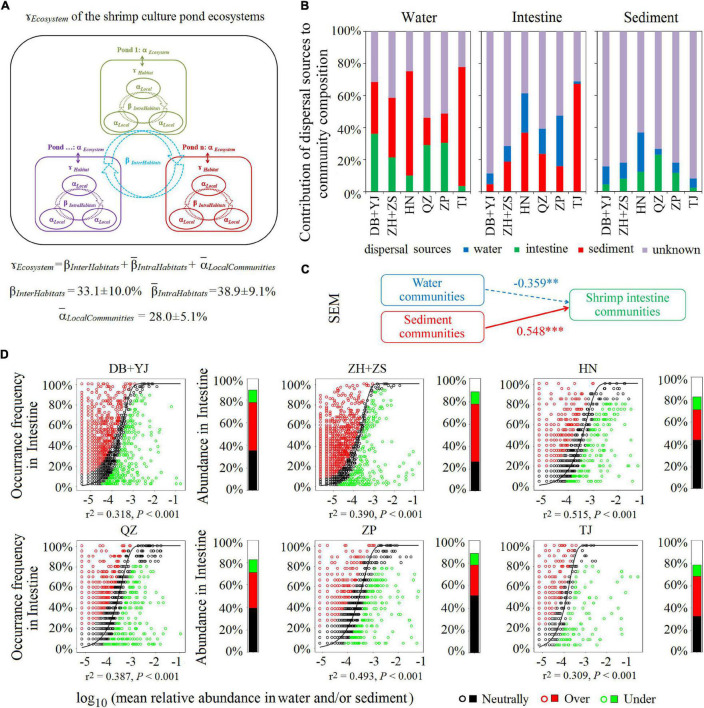
The contribution of microbial communities in water, shrimp intestine, and sediment habitats to the SCPE metacommunity of six regions. **(A)** Multiscale hierarchical partitioning of microbial diversity. **(B)** SourceTracker analysis of contributions of water, shrimp intestine, and sediment source communities to each other’s communities. **(C)** The SEM shows environmental community drivers of shrimp intestinal microbiota in the SCPE. The directed graph of SEM, and the goodness-of-fit (GoF) statistic value was 0.626. Each box represents an observed variable or latent variable. Path coefficients are reflected in the width of the arrow with solid and dashed arrows indicating significantly positive and negative effects, respectively. ^∗∗∗^: *P* < 0.001, ^∗∗^: *P* < 0.01. **(D)** The Sloan neutral model applied to shrimp intestine communities with their corresponding surrounding water and/or sediment communities as the sources. Stacked bar chart depicts the relative abundance of sequences in the neutrally distributed (black), overrepresented (red), and underrepresented (green) OTUs in shrimp intestine.

To evaluate the contribution of each of the three habitats (water, sediment, and shrimp intestine) to the regional diversity of the SCPE metacommunity, we used additive partitioning of diversity from local to regional scales. We examined whether the microbial diversity observed at the ecosystem level (Ɣ_*Ecosystem*_) was primarily due to a high microbial dissimilarity among ponds (β_*InterHabitats*_), a high dissimilarity among communities within each pond ($\bar{\beta }$_*IntraHabitats*_), or from a high microbial diversity within each local community ($\bar{\alpha }$_*LocalCommunities*_, i.e., water, shrimp intestine, or sediment sample). The results show that the contribution of $\bar{\alpha }$_*LocalCommunities*_ to the metacommunity diversity (Ɣ_*Ecosystem*_) was 28.0% ± 5.1%, inferior to β_*InterHabitats*_ (33.1% ± 10.0%) and $\bar{\beta }$_*IntraHabitats*_ (38.9% ± 9.1%) in their contributions to Ɣ_*Ecosystem*_ (Figure 3A). The results reveal that $\bar{\beta }$_*IntraHabitats*_ and β_*InterHabitats*_ were important for generating the microbial diversity in the SCPE.

To test if the microbial communities of environmental water and sediment have a decisive role in shrimp intestinal microbiota, we evaluated the contribution of different source communities to the three habitats in the SCPE in six regions by SourceTracker. For shrimp intestinal communities, the most dominant potential source was sediment (an average of 27.8%), followed by water (15.2%); for water communities, the most dominant potential source was also sediment (40.6%), followed by shrimp intestine (22.3%) (Figure 3B). For sediment communities, water or shrimp intestine only contributed about 10% each (Figure 3B). These results indicate that the microbial communities of each habitat in the SCPE could be a source for the other two. Although sediment appeared to be the most important source for both water and shrimp intestinal communities, more importantly, both water and sediment microbial communities contributed to the shrimp intestinal microbiota in the SCPE. The contribution of water and sediment communities to the shrimp intestinal microbiota was corroborated by the results of SEM analysis (Figure 3C).

The Sloan neutral community model was further applied to analyze the shared OTUs between the environmental water/or sediment and shrimp intestine samples. That is, neutral distribution (black points) accounted for 38.2% ± 8.5% in water and/or sediment microbial communities of six regions, and the proportions of overrepresented (red points) and underrepresented (green points) OTUs were 35.8% ± 9.6% and 11.0% ± 1.1%, respectively (Figure 3D). Thus, the proportion of shared and neutrally distributed OTUs between shrimp intestine and water and/or sediment was relatively high, suggesting that a significant proportion of microbial communities of shrimp intestine tended to colonize from surrounding environments.

### Ecological Processes Governing the Microbial Assembly of the Three Shrimp Culture Pond Ecosystem Habitats in Six Regions

**FIGURE 4:**
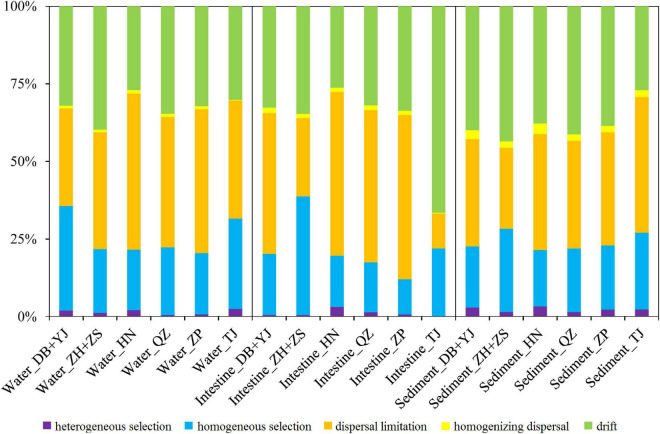
The contribution of ecological processes on the microbial assembly of water, shrimp intestine, and sediment habitats of six regions.

To understand the microbial assembly mechanisms at play in the three SCPE habitats in six regions, we quantified the relative contribution of major ecological processes that structure the microbiota using iCAMP. The results show that most of the microbial variation was controlled by dispersal limitation (35–40%) and drift (30–40%) (Figure 4). Thus, stochastic factors appear to be more important in influencing the microbial assembly of the three SCPE habitats at a regional scale. Also, homogeneous selection contributed 20–25% of the microbial variation (Figure 4). Additionally, we also used SEM analysis to reveal the effect of environmental drivers on water and sediment microbiota and found that water (*r* = 0.406, *P* < 0.001) and sediment properties (*r* = 0.579, *P* < 0.001) significantly affected their microbial community structure (Supplementary Figure 5).

### Environmental Water and Sediment Microbiota Contributed to Community Succession of the Shrimp Intestinal Microbiota in the Shrimp Culture Pond Ecosystem Across Different Shrimp Culture Developmental Stages

**FIGURE 5:**
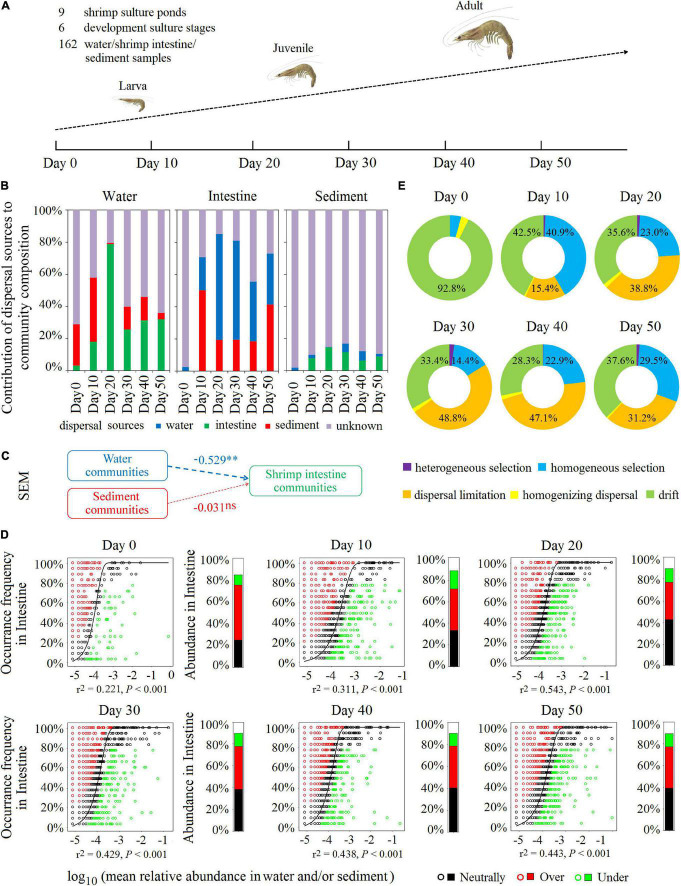
Environmental water and sediment microbiota contributed to community succession of the shrimp intestinal microbiota in the SCPE during shrimp developmental stages. **(A)** Nine shrimp culture ponds were selected in the study area, which was located at Zhuhai in China. Water, shrimp intestine, and sediment samples with nine samples each for each time at six developmental stages: day 0, day 10, day 20, day 30, day 40, and day 50 post-larval shrimp inoculation. Microbial communities of the surrounding water and sediment mainly contributed to shrimp intestinal microbiota in different culture stages. **(B)** SourceTracker analysis of the contribution of water, shrimp intestine, and sediment source communities to each other’s communities. **(C)** The SEM shows environmental microbial community drivers of shrimp intestinal microbiota in the SCPE. The directed graph of SEM and the GoF statistic value was 0.370. Each box represents an observed or latent variable. Path coefficients are reflected in the width of the arrow with dashed arrows as significantly negative effects. ^∗∗^: *P* < 0.01, ns: no significance. **(D)** The Sloan neutral model was applied to shrimp intestine communities with their corresponding surrounding water and/or sediment communities as the sources. Stacked bar chart depicts the relative abundance of sequences in the neutrally distributed (black), overrepresented (red), and underrepresented (green) OTUs in shrimp intestine. **(E)** The contribution of ecological processes to the microbial assembly of shrimp intestine habitat.

In the experiment to further verify the contribution of the environmental water and sediment microbial communities to shrimp intestinal microbiota, we analyzed the dynamics of microbial communities from three habitats across six cultural developmental stages: day 0, day 10, day 20, day 30, day 40, and day 50 with similar ecological features being present at each of the six regional sites (Figure 5A). Microbial diversity (based on OTU number, Shannon index, and Chao 1 index) was the highest in the sediment, followed by water and shrimp intestine for all SCPEs examined and at each of the six developmental stages (Supplementary Table 14 and Supplementary Figure 6A). Both NMDS and ANOSIM analyses showed that the microbial community structure significantly (*P* < 0.001) differed between any two of compared habitats at each culture developmental stage (Supplementary Figure 6B). Similar to the results of the six regions, most OTUs were present in the sediment habitat, and a high percentage (∼80%) of OTUs in the shrimp intestine and water habitats were shared with the sediment habitat at six cultural development stages (Supplementary Figure 7). Similar trends in microbial compositions of the three habitats at each of the six cultural development stages were observed in each pond (Supplementary Figure 8). We also compared microbial co-association networks of the three habitats at each of the six cultural development stages. The microbial networks in the sediment and shrimp intestines were more complex and better connected than that in the water habitat, and OTU31337, OTU433, and OTU13771 were the keystone species in the water, shrimp intestine, and sediment habitats, respectively (Supplementary Figure 9 and Supplementary Tables 15, 16).

Consistent with the results of the six regions, SourceTracker analysis also indicates that, at each cultural development stage, any of the habitat microbial communities could be a source for the other two communities. The most dominant potential source for the water microbial communities was from shrimp intestine and sediment, but for the sediment microbial communities, only ∼12% was attributable to the water and shrimp intestine (Figure 5B). Whereas for the shrimp intestinal microbial communities, water and sediment microbiota were the dominant potential sources, accounting for up to 69.9–84.7% (days 10–50) (Figure 5B), suggesting that environmental microbial communities were important sources for the establishment of the shrimp intestinal microbiota. On days 10 and 50, sediment represented almost half of the potential source for shrimp intestinal microbiota although on days 20, 30, and 40, water was the dominant source (Figure 5B). These results were further supported by results of the SEM and Sloan neutral community model analyses (Figures 5C,D).

Additionally, during the six cultural development stages in the SCPE, stochastic factors were the dominant microbial assembly mechanism for all three habitats (Figure 5E and Supplementary Figures 10A,B), specifically dispersal limitation (20–30%), drift (30–55%), and homogeneous selection (20–30%). SEM analysis shows that water (*r* = 0.370, *P* < 0.001) and sediment properties (*r* = 0.641, *P* < 0.001) significantly affect the associated microbial structure (Supplementary Figures 10C,D). More importantly, combined with the results of SourceTracker, SEM, and Sloan neutral community model analyses, these results indicate that environmental microbial communities had a large contribution to the succession of shrimp intestinal microbiota in the SCPE metacommunity, which may be due to the relatively highly stochastic processes involved in the microbial assembly of the shrimp intestine.

## Discussion

The intimate link among environment, the intestinal microbiota, and host health status is highly concerned with human and animal health (Wall et al., 2015). The microbial communities from the multiple habitats within an aquatic ecosystem constitute the aquatic metacommunity, but how environmental microbial communities shape the shrimp intestinal microbiota has not been well studied. In this study, we considered microbial communities of multiple habitats as the metacommunity in aquatic ecosystems and analyzed the microbial assembly of water, shrimp intestine, and sediment habitats. Our results show that core microbial taxa were in each habitat and the SCPE metacommunity (H1) and that environmental water and sediment communities dominated the shrimp intestinal microecosystem; moreover, microbial assembly of three habitats in the SCPE appeared to be largely driven by stochastic processes (H2), which generally supports our hypothesis.

Core microbial taxa provide information on putatively important microorganisms for ecosystem functioning (Saunders et al., 2016). Previous studies identify several bacterial OTUs as core taxa in soil (9), human feces (6), air (2), fresh water (1), and wastewater treatment plants (2) (Wu et al., 2019), suggesting that various ecosystems may have different core populations, possibly due to the number of samples used or the high dissimilarity of the environments examined. In this study, the core microbial OTUs were distinct in each habitat. There was an overlap of 23 core OTUs from shrimp intestine that were also present in the water and/or sediment habitats, which is generally consistent with previous studies. For example, the surrounding environments are shown to be a source of microbial species colonizing aquatic animal intestine and vice versa (Cahill, 2004; Sullam et al., 2012). Another study suggests that the microbes colonizing shrimp intestines are selected from the surrounding environments to improve host fitness (Xiong et al., 2018). It is noteworthy that some core taxa detected in this study are derived from some known shrimp opportunistic pathogens, including *Photobacterium* OTU3557, *Vibrio* OTU1384, *Vibrio* OTU1482, *Vibrio* OTU2357, *Vibrio* OTU2482, and *Candidatus* Bacilloplasma OTU1192. An increased abundance of these species in shrimp intestines is generally associated with disease outbreaks (Xiong et al., 2017, 2018; Hou et al., 2018). Moreover, the core taxa of water and sediment habitats in the SCPE may associate with their known biological functions. For example, several core OTUs belonged to *Rhodobacter*, and some oxygenic photosynthetic microbes are known to enhance carbon cycling and energy capture from sunlight in aquatic ecosystems (Chang et al., 1991). A *Truepera* OTU (OTU15485) was identified as a core taxon in an aquatic sediment habitat, reflecting the importance of this species in organic matter degradation in aquatic ecosystems (Albuquerque et al., 2005). Thus, core microbial taxa were among the three habitats across the SCPE or in each habitat, indicating such core taxa in shrimp intestine may play key roles for shrimp and environment health in the SCPE.

Generally, multiple habitats constitute a metacommunity for the overall microbial diversity in aquatic ecosystems (Zeng et al., 2017), surrounding environments are the main sources of microbes colonizing aquatic animal intestines, and the host animal drives, in a large part, the selection of microorganisms (Bolnick et al., 2014). Theoretically, aquatic animals are microorganism-free at birth, so postnatally acquired intestinal microorganisms should immigrate from their surroundings (Yan et al., 2016). As all activities carried out by aquatic animals (e.g., feeding and defecation) take place in the surrounding water or/and sediment habitats, interactions between a host and its environment are more direct than with terrestrial animals and their environments; thus, the assembly of aquatic animal intestinal microbial communities is directly influenced by the microbes present in the surrounding environment (Bolnick et al., 2014). Consistently, Cahill considers that the bacteria present in aquatic environments influence the composition of animal intestinal microbial communities (Cahill, 2004). Sullam et al. (2012) found that fish could acquire intestinal bacteria through water cyclic transmission by which hosts obtained their bacterial communities from their environments. Our results reveal that microbial communities from the water, shrimp intestine, and sediment habitats in the SCPE had close relationships as these three habitats are connected to each other by various biological and ecological processes, including nutrient sharing, dispersal, and microbial interactions (Hubbell, 2001; Schryver and Vadstein, 2014; Adair and Douglas, 2017). For instance, dispersion is a key factor influencing the metacommunity and its associated community structure (Fodelianakis et al., 2019). In this study, we observed a high percentage of dispersion from sediment to the other two habitats in the SCPE. Compared with water communities, shrimp intestine communities were more closely related to sediment communities. This is likely due to sediment features and the shrimp’s lifestyles. It is well known that *L. vannamei* is planktobenthos (mainly living in the benthic zone and occasionally floating in the water) and is most often active in the sediment habitat. Also, *L. vannamei* has the characteristic of feeding from the sediment and ingestion of particulate matter into its intestine. Our results indicate that water and sediment communities mainly contribute to shrimp intestinal microbiota, but such mechanisms need to be further investigated.

We also found that each habitat harbored distinct microbial communities, indicating that different taxa have obvious preferences in three habitats. For example, *Vibrio*, *Photobacterium*, and *Candidatus* Bacilloplasma were enriched in the shrimp intestine. These genera are known opportunistic pathogens and are widespread in cultural pond ecosystems (Vadstein et al., 2004; Li et al., 2017; Xiong et al., 2017). The aquatic animal intestine may offer a more favorable microenvironment for such taxa, allowing them to become dominant in the shrimp intestine. They may then further spread into their surrounding environments through the excretion of aquatic animals, making the control of opportunistic pathogen proliferation extremely challenging (Tania et al., 2018). For example, pathogens may be reintroduced to the SCPE through excrement “seeds” after treatment with disinfectant (Gustafson and Bowen, 1997). As aquatic animals are important for maintaining microbial diversity in aquatic environments (Troussellier et al., 2017), microbial ecological management strategies are needed to inhibit opportunistic pathogens in aquaculture ecosystems.

Examination of underlying microbial assembly mechanisms shows that stochastic processes play a more important role in influencing the microbial community structure than deterministic processes. A possible explanation is that ecological drift (e.g., stochastic processes of birth, death, colonization) becomes stronger due to high dispersal rates (Zhou et al., 2014). A recent global-scale study of activated sludge communities from wastewater treatment plants indicates that microbial spatial turnover is largely driven by stochastic processes (Wu et al., 2019). Similarly, in other natural and engineered ecosystems, such as temperate forest (Bahram et al., 2016), grasslands (under warming conditions) (Guo et al., 2018), bioreactors (Zhou et al., 2013), and groundwater systems (perturbed by adding emulsified vegetable oil for uranium immobilization) (Zhou et al., 2014), stochastic processes played larger roles than deterministic ones in explaining the microbial assembly. Our present study is largely consistent with those previous studies, suggesting that the microbial assembly was largely driven by stochastic processes in the SCPE. Moreover, across different shrimp cultural development stages, the microbial communities in each SCPE habitat consistently displayed similar dynamics variation. Water and sediment microbial communities make important contributions to the shrimp intestinal microbiota at a temporal scale, which may indicate that the microbial succession of the shrimp intestinal community is shaped by the environmental water and sediment microbial communities.

## Conclusion

**FIGURE 6:**
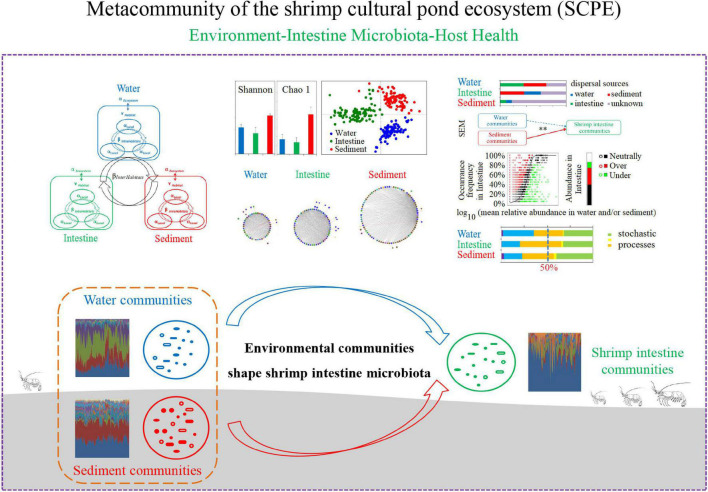
A schematic presentation of microbial assembly mechanisms and relationships among microbial communities of water, shrimp intestine, and sediment habitats in the SCPE metacommunity.

In summary, we systematically evaluated the microbial community composition of water, shrimp intestine, and sediment habitats in the SCPE as a metacommunity and revealed their relationships among the environment, the intestine microbiota and host health status, and possible assembly mechanisms (Figure 6). Specifically, we identified core microbial taxa for each habitat and the metacommunity, determined that environmental water and sediment communities dominated the intestinal microecosystem of *L. vannamei*, and found that microbial variation was largely controlled by stochastic processes in the SCPE. These findings enrich our understanding of the environment–intestinal microbiota–host health closely linked relationship, making it possible to be the central dogma for an anthropogenic aquaculture ecosystem. This study provides new insights into microbial assembly mechanisms and a framework for metacommunity analysis in the SCPE and has important implications for developing new strategies for animal health.

## Data Availability Statement

The datasets presented in this study can be found in online repositories. The names of the repository/repositories and accession number(s) can be found in the article/Supplementary Material.

## Author Contributions

All authors contributed to experimental assistance and intellectual input to this study. ZJH, JH, ZLH, and DH conceived the original concept. ZJH, DH, ZLH, and JH developed the experimental strategies and sampling design. DH, ZJH, RZ, SZ, CGX, DW, XD, LY, HW, ZD, SW, DN, CLX, QY, JZ, ZLH, and JH performed the sample collections, DNA extraction, DNA sequencing and data analyses. ZJH, DH, JZ, ZLH, and JH worte the manuscript.

## Conflict of Interest

The authors declare that the research was conducted in the absence of any commercial or financial relationships that could be construed as a potential conflict of interest.

## Publisher’s Note

All claims expressed in this article are solely those of the authors and do not necessarily represent those of their affiliated organizations, or those of the publisher, the editors and the reviewers. Any product that may be evaluated in this article, or claim that may be made by its manufacturer, is not guaranteed or endorsed by the publisher.

## Footnotes

1
https://cytoscape.org/
