# Supplementary material for: Environmental Water and Sediment Microbial Communities Shape Intestine Microbiota for Host Health: The Central Dogma in an Anthropogenic Aquaculture Ecosystem
Source: Front Microbiol. 2021 Nov 2;12:772149. doi: 10.3389/fmicb.2021.772149 (PMC8593368; doi:10.3389/fmicb.2021.772149)
Supplement: Supplementary file 1 [file Data_Sheet_1.ZIP › Supplementary Materials/Supplementary Materials.docx]

Supplementary Materials for

**Environmental water and sediment microbial communities shape intestinal microbiota for host health: the central dogma in anthropogenic aquaculture ecosystem**

Zhijian Huang, Dongwei Hou, Renjun Zhou, Shenzheng Zeng, Chengguang Xing, Dongdong Wei, Xisha Deng, Lingfei Yu, Hao Wang, Zhixuan Deng, Shaoping Weng, Daliang Ning, Qingyun Yan, Jizhong Zhou, Zhili He, Jianguo He

Correspondence to: Dr. Zhijian Huang, lsshzhj@mail.sysu.edu.cn;

Dr. Zhili He, hezhili@mail.sysu.edu.cn;

Dr. Jianguo He, lsshjg@mail.sysu.edu.cn

**This file includes:**

Figs. S1 to S10

Tables S1 to S16

**Supplementary Figures**


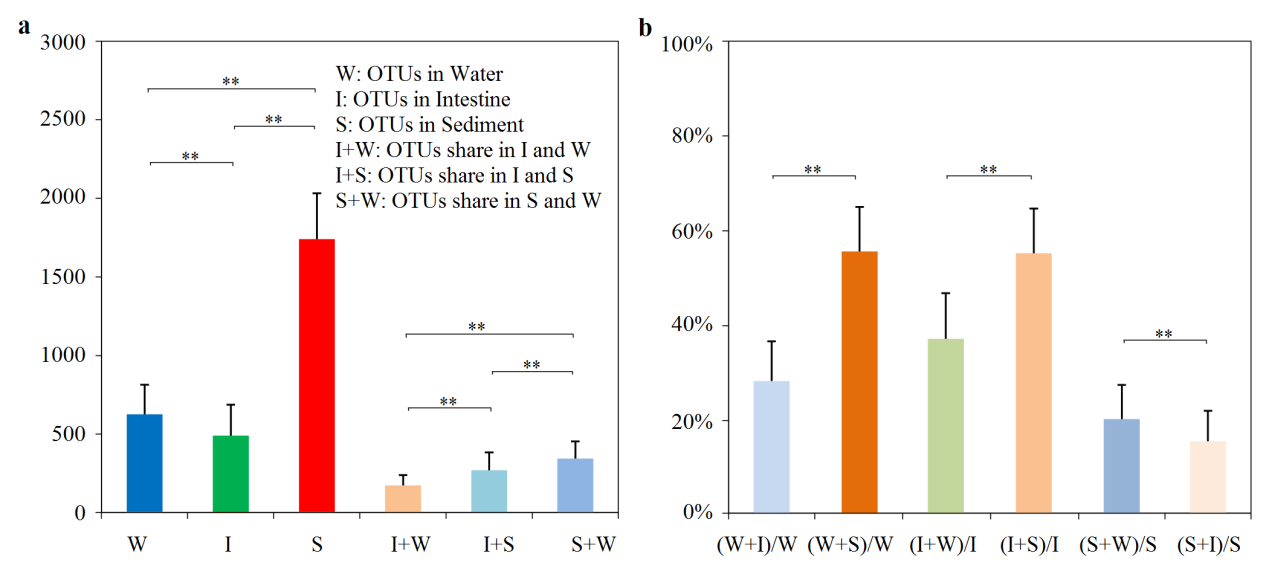


**FIGURE S1. Schematic presentation of detected OTUs found in water, shrimp intestine, and sediment habitats of each pond by Venn analysis of six regional sites.** (a) The numbers of OTUs in each habitat and shared in two or three habitats. (b) The percentage of OTUs shared in one with other habitat in this habitat. Welch’s *t*-test analysis showed that the OTU numbers or percentage of OTUs were significantly different between any two of the compared habitats. (**: *P* < 0.01)


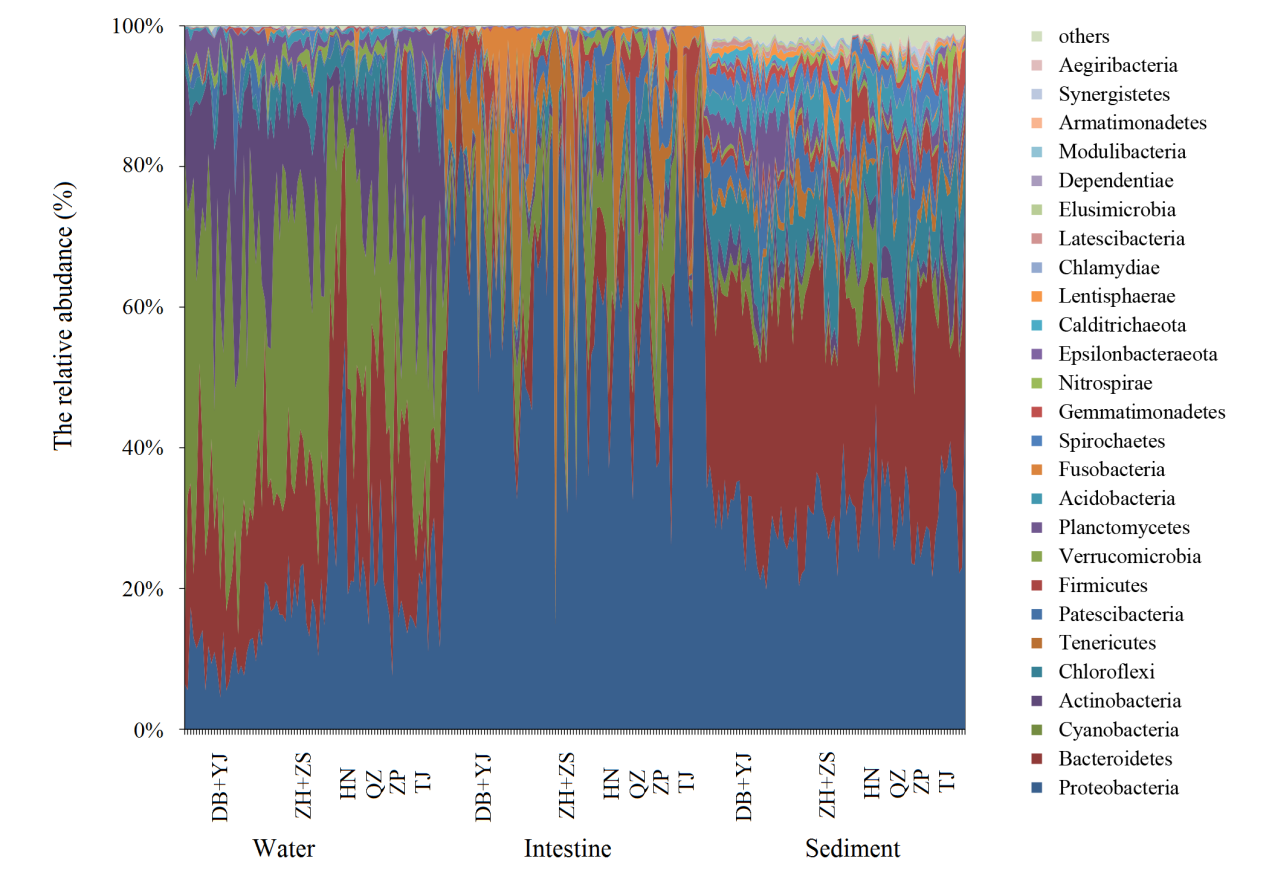


**FIGURE S2. The relative abundance of phyla from water, shrimp intestine, and sediment samples in each pond of six regional sites.**


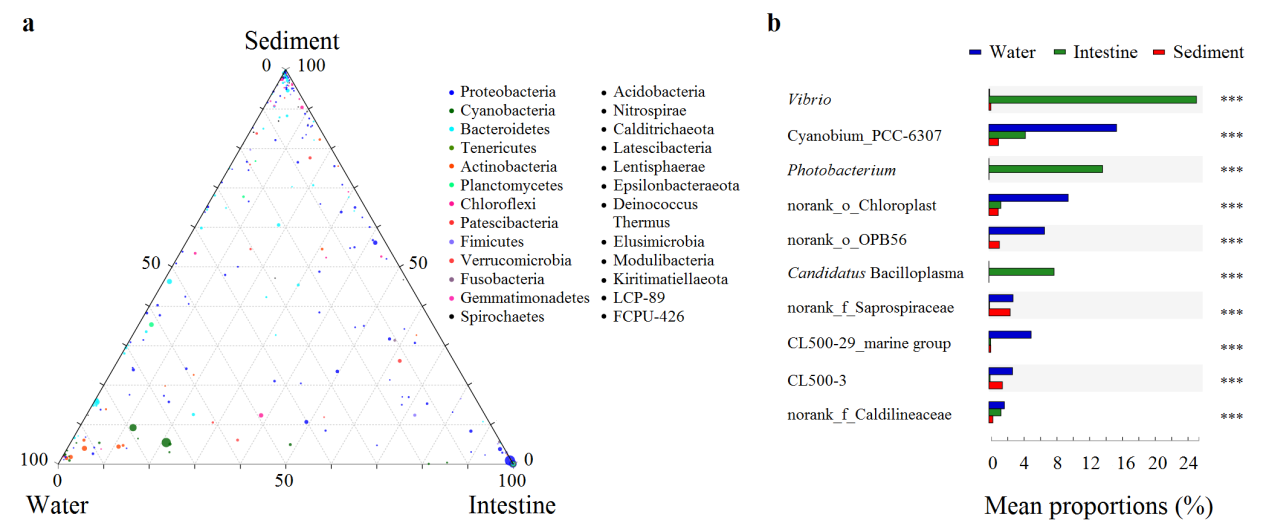


**FIGURE S3. Comparisons of microbial community compositions of water, shrimp intestine, and sediment habitats in the SCPE.** (a) The ternary plot showed percentages and relative abundances of genera (> 0.1%) among three habitats, and that each habitat harbored unique assemblages, and different genera had preferences in three habitats. (b) Microbial taxa responsible for the difference among three habitats at the genus level as determined by one-way ANOVA (***: *P* < 0.001). The results showed that the relative abundance of many genera differed significantly in three habitats (only the relative abundances of most ten genera are shown, and see the Supplementary Table S7 for details). For example, *Vibrio*, *Photobacterium* and *Candidatus* Bacilloplasma were present in three habitats, but predominant in the shrimp intestine.

**
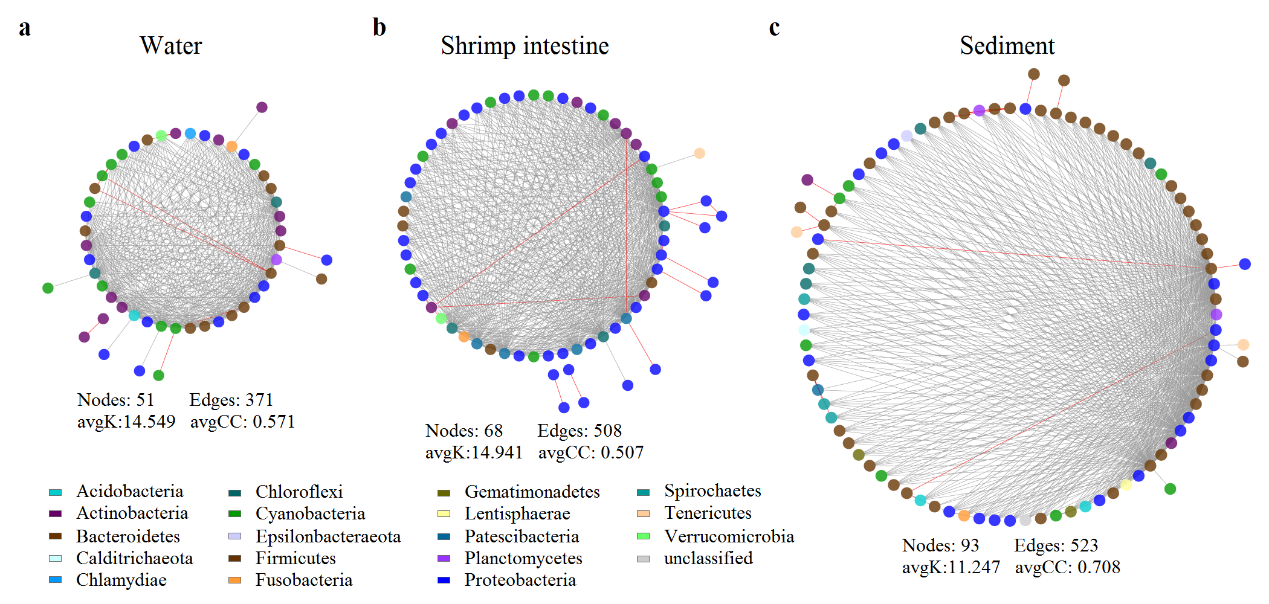
**

**FIGURE S4. Microbial co-association network in the SCPE at six regional sites.** The microbial interspecies interactions of water (a), shrimp intestine (b) and sediment (c) habitats. Each node represents a microbial OTU. The colours of nodes indicate OTUs affiliated to different phyla. A *red edge* indicates positive interaction between two individual nodes, whereas a *gray edge* indicates negative interaction.

**
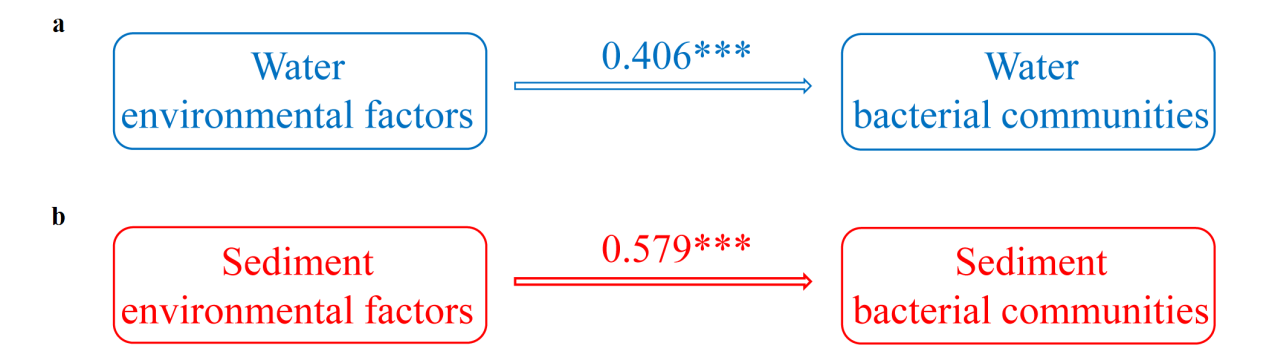
**

**FIGURE S5. The SEM shows environmental drivers of water (a) and sediment (b) microbiota in the SCPE.** The directed graph of SEM, and the Goodness-of-Fit (GoF) statistic values were 0.262 and 0.453. Each box represents an observed variable or latent variable. Path coefﬁcients are reﬂected in the width of arrow, with solid arrows indicating signiﬁcantly positive effects. ***: *P* < 0.001


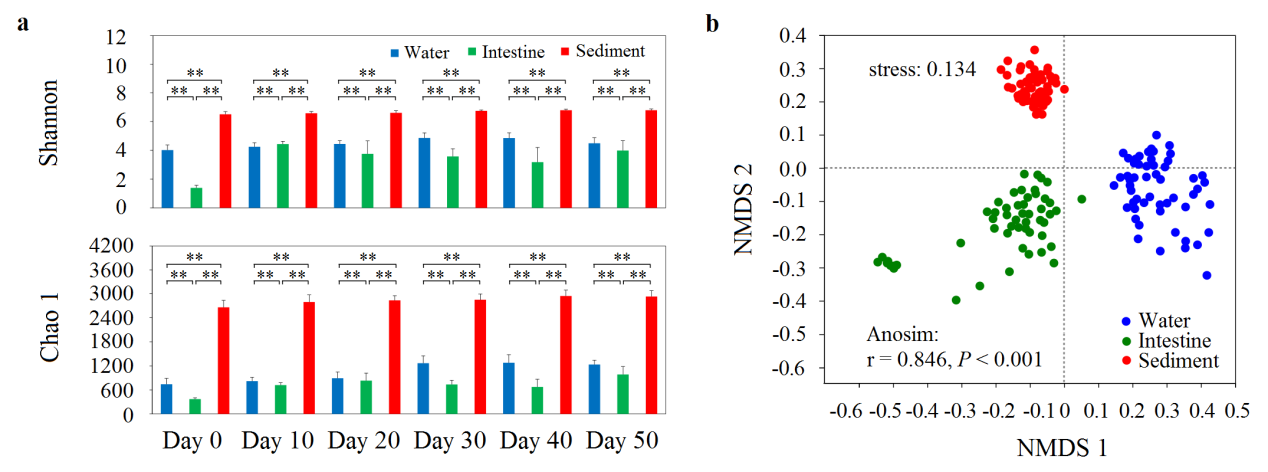


**FIGURE S6. Microbial diversity among water, shrimp intestine and sediment habitats in six culture stages.** (a) Statistical significance of the α-diversity indices among three habitats were based on the Welch’s *t*-test (**: *P* < 0.01). (b) β-diversity of microbial communities of three habitats analyzed by NMDS and ANOSIM based on the Bray-Curtis distance.


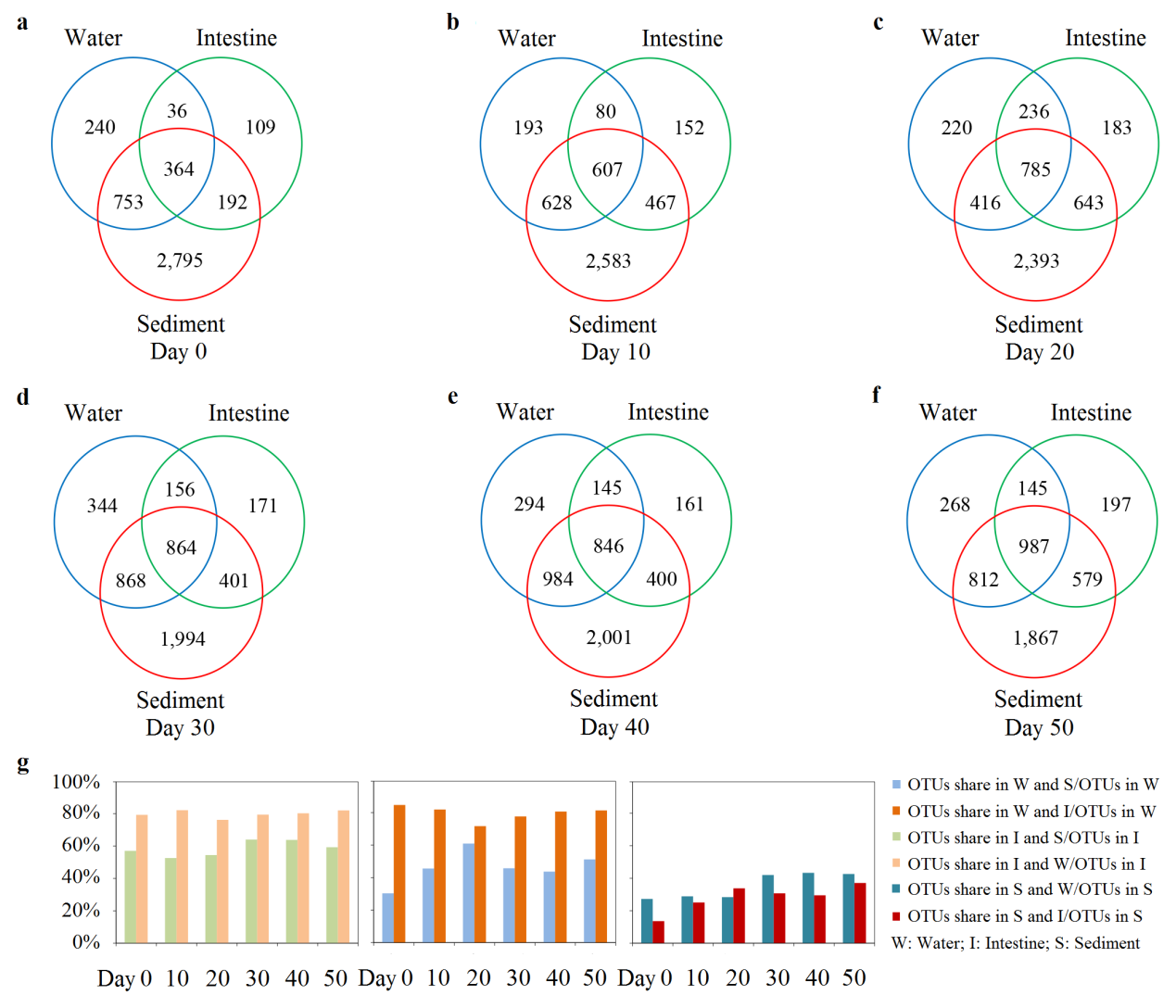


**FIGURE S7. Venn analysis of microbial community compositions in water, shrimp intestine and sediment habitats based on detected OTUs in six culture stages.** (a-f) The numbers of OTUs in each habitat and shared in two or three habitats. (g) The percentage of OTUs shared in one with other habitat in this habitat.


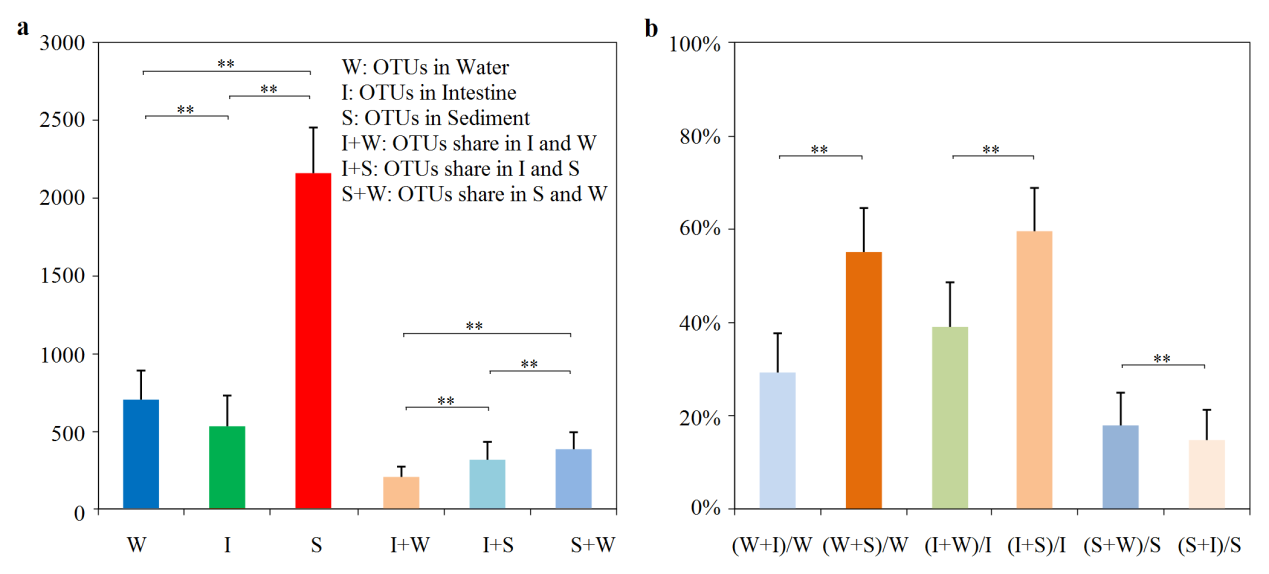


**FIGURE S8. Schematic presentation of detected OTUs found in water, shrimp intestine, and sediment habitats of each pond by Venn analysis in six culture stages.** (a) The numbers of OTUs in each habitat and shared in two or three habitats. (b) The percentage of OTUs shared in one with other habitat in this habitat. Welch’s *t*-test analysis showed that the OTU numbers or percentage of OTUs were significantly different between any two of the compared habitats. (**: *P* < 0.01)

**
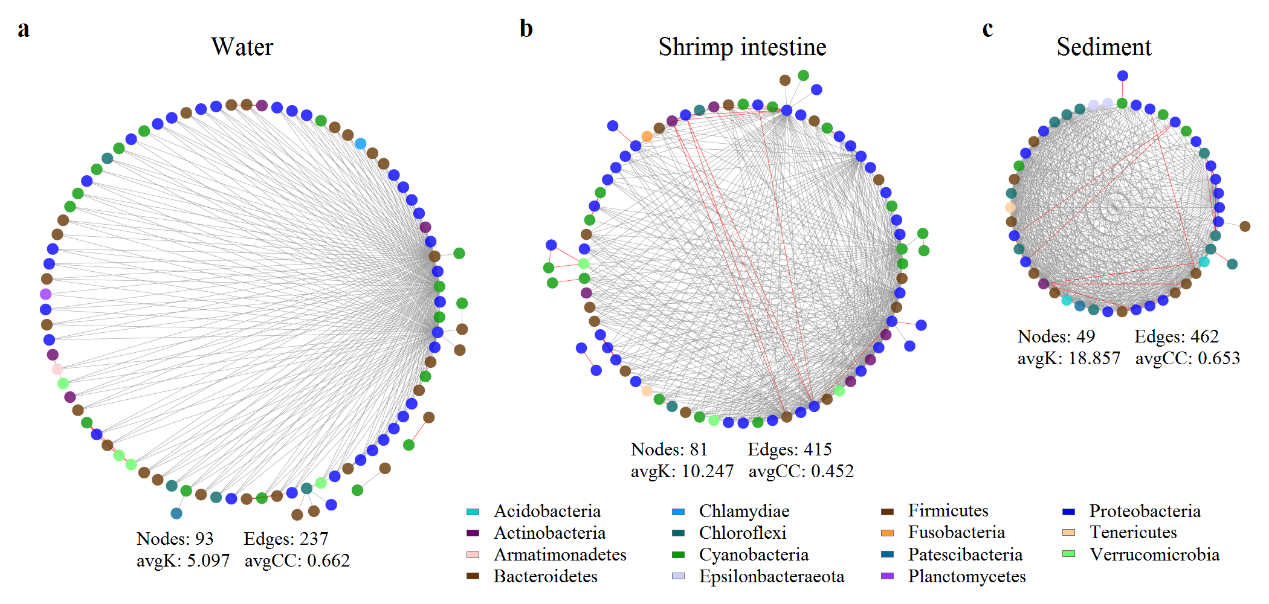
 FIGURE S9. Microbial co-association network in the SCPE at six culture developmental stages.** The microbial interspecies interactions of water (a), shrimp intestine (b) and sediment (c) habitats. Each node represents a microbial OTU. The colours of nodes indicate OTUs affiliated to different phyla. A *red edge* indicates positive interaction between two individual nodes, whereas a *gray edge* indicates negative interaction.

**
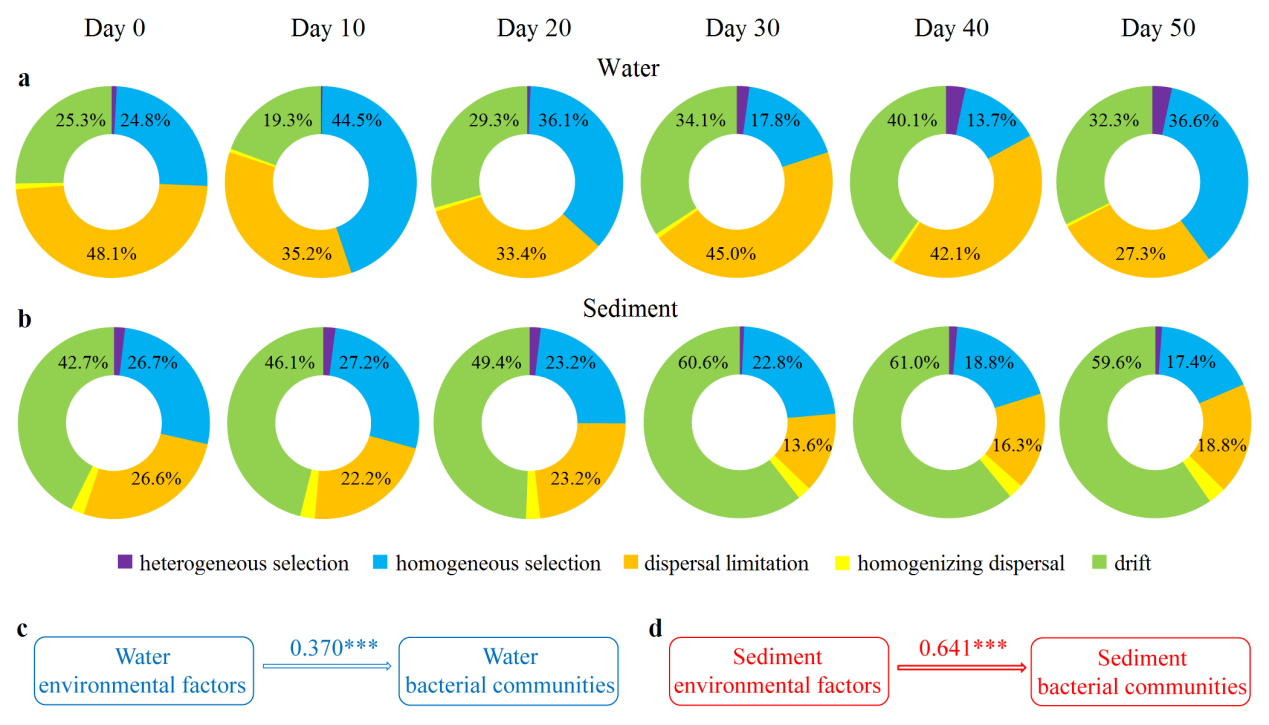
**

**FIGURE S10. The contribution of ecological processes on the microbial assembly of water (a) and sediment (b) habitats in six culture stages. (c,d) The SEM shows environmental drivers of water and sediment microbiota in the SCPE.** The directed graph of SEM, and the Goodness-of-Fit (GoF) statistic values were 0.262 and 0.453. Each box represents an observed variable or latent variable. Path coefﬁcients are reﬂected in the width of arrow, with solid arrows indicating signiﬁcantly positive effects. ***: *P* < 0.001

**Supplementary Tables**

**Table S1 Characteristics of sampling sites of six regional sites**

See the excel file.

**Table S2** **Environmental factors of water samples collected in shrimp cultural ponds of six regional sites**

See the excel file.

**Table S3 Environmental factors of sediment samples collected in shrimp cultural ponds of six regional sites**

See the excel file.

**Table S4 Characteristics of sampling sites of six culture stages**

| Sample ID | Province | City | Date (mm/dd/yyyy) | Latitude (◦N) | Longitude (◦E) | Area (m^2^) |
| --- | --- | --- | --- | --- | --- | --- |
| Water-Day0 | Guangdong | Zhuhai | 06/21/2018 | 22.3681 | 113.2208 | ≈ 3,300 |
| Water-Day10 |  |  | 07/01/2018 | 22.3681 | 113.2206 | ≈ 3,300 |
| Water-Day20 |  |  | 07/11/2018 | 22.3681 | 113.2206 | ≈ 3,300 |
| Water-Day30 |  |  | 07/21/2018 | 22.3683 | 113.2206 | ≈ 3,300 |
| Water-Day40 |  |  | 07/31/2018 | 22.3681 | 113.2206 | ≈ 3,300 |
| Water-Day50 |  |  | 08/10/2018 | 22.3683 | 113.2208 | ≈ 3,300 |
| Intestine-Day0 |  |  | 06/21/2018 | 22.3681 | 113.2208 | ≈ 3,300 |
| Intestine-Day10 |  |  | 07/01/2018 | 22.3681 | 113.2206 | ≈ 3,300 |
| Intestine-Day20 |  |  | 07/11/2018 | 22.3681 | 113.2206 | ≈ 3,300 |
| Intestine-Day30 |  |  | 07/21/2018 | 22.3683 | 113.2206 | ≈ 3,300 |
| Intestine-Day40 |  |  | 07/31/2018 | 22.3681 | 113.2206 | ≈ 3,300 |
| Intestine-Day50 |  |  | 08/10/2018 | 22.3683 | 113.2208 | ≈ 3,300 |
| Sediment-Day0 |  |  | 06/21/2018 | 22.3681 | 113.2208 | ≈ 3,300 |
| Sediment-Day10 |  |  | 07/01/2018 | 22.3681 | 113.2206 | ≈ 3,300 |
| Sediment-Day20 |  |  | 07/11/2018 | 22.3681 | 113.2206 | ≈ 3,300 |
| Sediment-Day30 |  |  | 07/21/2018 | 22.3683 | 113.2206 | ≈ 3,300 |
| Sediment-Day40 |  |  | 07/31/2018 | 22.3681 | 113.2206 | ≈ 3,300 |
| Sediment-Day50 |  |  | 08/10/2018 | 22.3683 | 113.2208 | ≈ 3,300 |

**Table S5 Environmental factors of water samples collected in shrimp cultural ponds of six culture stages**

See the excel file.

**Table S6 Environmental factors of sediment samples collected in shrimp cultural ponds of six culture stages**

See the excel file.

**Table S7 Summary of OTU numbers and microbial diversity indices for water, shrimp intestine and sediment habitats of six regional sites**

| Sample | OTU numbers | Shannon | | Chao 1 | | Coverage | |
| --- | --- | --- | --- | --- | --- | --- | --- |
|  |  | mean | sd | mean | sd | mean | sd |
| All | 7,656 | / | / | / | / | / | / |
| Water | 5,078 | 4.37 | 0.50 | 851 | 276 | 0.98 | 0.01 |
| Intestine | 3,919 | 3.39 | 0.99 | 680 | 259 | 0.99 | 0.00 |
| Sediment | 7,389 | 6.28 | 0.28 | 2,273 | 411 | 0.96 | 0.01 |

**Table S8** **The relative abundance of core OTUs from water, shrimp intestine and sediment habitats, and the taxonomic composition of core OTUs at the phylum and genus levels**

See the excel file.

**Table S9** **The relative abundance of genera from water, shrimp intestine, and sediment samples in each pond of six regional sites**

See the excel file.

**Table S10 Taxa responsible for differences among water, shrimp intestine and sediment habitats at phylum level as determined by one-way ANOVA**

See the excel file.

**Table S11 Taxa responsible for differences among water, shrimp intestine and sediment habitats at genus level as determined by one-way ANOVA**

See the excel file.

**Table S12 Topological properties of ecology network on the microbial communities of water, shrimp intestine and sediment habitats in the SCPE at six regional sites**

|  | Water | Intestine | Sediment |
| --- | --- | --- | --- |
| Total nodes | 51 | 68 | 93 |
| Total links | 371 | 508 | 523 |
| R square of power-law | 0.029 | 0.068 | 0.23 |
| Average degree (avgK) | 14.549 | 14.941 | 11.247 |
| Average clustering coefficient (avgCC) | 0.571 | 0.507 | 0.708 |
| Average path distance (GD) | 1.873 | 1.887 | 2.025 |
| Geodesic efficiency (E) | 0.627 | 0.603 | 0.537 |
| Harmonic geodesic distance (HD) | 1.594 | 1.657 | 1.862 |
| Maximal degree | 37 | 50 | 79 |
| Nodes with max degree | OTU4327; OTU16092 | OTU9882 | OTU16554 |
| Centralization of degree (CD) | 0.467 | 0.539 | 0.753 |
| Maximal betweenness | 112.356 | 333.143 | 706.543 |
| Nodes with max betweenness | OTU4327 | OTU9882 | OTU16554 |
| Centralization of betweenness (CB) | 0.077 | 0.141 | 0.159 |
| Maximal stress centrality | 758 | 1320 | 3468 |
| Nodes with max stress centrality | OTU4327 | OTU9882 | OTU16554 |
| Centralization of stress centrality (CS) | 0.461 | 0.482 | 0.768 |
| Maximal eigenvector centrality | 0.24 | 0.242 | 0.296 |
| Nodes with max eigenvector centrality | OTU16092 | OTU9882 | OTU16554 |
| Centralization of eigenvector centrality (CE) | 0.126 | 0.146 | 0.216 |
| Density (D) | 0.291 | 0.223 | 0.122 |
| Reciprocity | 1 | 1 | 1 |
| Transitivity (Trans) | 0.537 | 0.403 | 0.198 |
| Connectedness (Con) | 0.923 | 0.886 | 1 |
| Efficiency | 0.699 | 0.761 | 0.887 |
| Hierarchy | 0 | 0 | 0 |
| Lubness | 1 | 1 | 1 |

**Table S13** **Network nodes’ centrality indices of microbial communities of water, shrimp intestine and sediment habitats in the SCPE at six regional sites**

See the excel file.

**Table S14 Summary of OTU numbers and microbial diversity indices for water, shrimp intestine and sediment habitats in six culture stages**

| Sample | OTU numbers | Shannon | | Chao 1 | | Coverage | |
| --- | --- | --- | --- | --- | --- | --- | --- |
|  |  | mean | sd | mean | sd | mean | sd |
| All | 5,856 | \ | \ | \ | \ |  |  |
| Water | 3,552 | \ | \ | \ | \ | 0.98 | 0.01 |
| Intestine | 2,796 | \ | \ | \ | \ | 0.98 | 0.01 |
| Sediment | 5,483 | \ | \ | \ | \ | 0.95 | 0.00 |
| Water-Day0 | 1,393 | 4.01 | 0.35 | 742 | 148 | \ | \ |
| Water-Day10 | 1,508 | 4.24 | 0.28 | 819 | 98 | \ | \ |
| Water-Day20 | 1,657 | 4.44 | 0.23 | 891 | 155 | \ | \ |
| Water-Day30 | 2,232 | 4.85 | 0.35 | 1,267 | 183 | \ | \ |
| Water-Day40 | 2,269 | 4.85 | 0.37 | 1,277 | 201 | \ | \ |
| Water-Day50 | 2,212 | 4.49 | 0.39 | 1,233 | 108 | \ | \ |
| Intestine-Day0 | 701 | 1.39 | 0.18 | 372 | 32 | \ | \ |
| Intestine-Day10 | 1,306 | 4.43 | 0.20 | 721 | 60 | \ | \ |
| Intestine-Day20 | 1,847 | 3.75 | 0.90 | 831 | 189 | \ | \ |
| Intestine-Day30 | 1,592 | 3.57 | 0.54 | 736 | 107 | \ | \ |
| Intestine-Day40 | 1,552 | 3.18 | 1.01 | 674 | 191 | \ | \ |
| Intestine-Day50 | 1,908 | 3.99 | 0.68 | 984 | 200 | \ | \ |
| Sediment-Day0 | 4,104 | 6.50 | 0.18 | 2,660 | 174 | \ | \ |
| Sediment-Day10 | 4,285 | 6.57 | 0.12 | 2,790 | 179 | \ | \ |
| Sediment-Day20 | 4,237 | 6.60 | 0.15 | 2,833 | 117 | \ | \ |
| Sediment-Day30 | 4,127 | 6.74 | 0.06 | 2,848 | 145 | \ | \ |
| Sediment-Day40 | 4,231 | 6.78 | 0.07 | 2,941 | 148 | \ | \ |
| Sediment-Day50 | 4,245 | 6.78 | 0.08 | 2,929 | 146 | \ | \ |

**Table S15 Topological properties of ecology network on the microbial communities of water, shrimp intestine and sediment in the SCPE at six culture stages**

|  | Water | Intestine | Sediment |
| --- | --- | --- | --- |
| Total nodes | 93 | 81 | 49 |
| Total links | 237 | 415 | 462 |
| R square of power-law | 0.789 | 0.633 | 0.02 |
| Average degree (avgK) | 5.097 | 10.247 | 18.857 |
| Average clustering coefficient (avgCC) | 0.662 | 0.452 | 0.653 |
| Average path distance (GD) | 2.255 | 2.127 | 1.718 |
| Geodesic efficiency (E) | 0.484 | 0.526 | 0.678 |
| Harmonic geodesic distance (HD) | 2.065 | 1.902 | 1.474 |
| Maximal degree | 74 | 55 | 42 |
| Nodes with max degree | OTU31337 | OTU433 | OTU13771 |
| Centralization of degree (CD) | 0.765 | 0.574 | 0.503 |
| Maximal betweenness | 1438.933 | 531.859 | 89.266 |
| Nodes with max betweenness | OTU31337 | OTU74 | OTU29373 |
| Centralization of betweenness (CB) | 0.335 | 0.157 | 0.065 |
| Maximal stress centrality | 4840 | 3017 | 595 |
| Nodes with max stress centrality | OTU31337 | OTU74 | OTU13771 |
| Centralization of stress centrality (CS) | 1.111 | 0.878 | 0.402 |
| Maximal eigenvector centrality | 0.43 | 0.305 | 0.237 |
| Nodes with max eigenvector centrality | OTU31337 | OTU433 | OTU13771 |
| Centralization of eigenvector centrality (CE) | 0.357 | 0.222 | 0.113 |
| Density (D) | 0.055 | 0.128 | 0.393 |
| Reciprocity | 1 | 1 | 1 |
| Transitivity (Trans) | 0.076 | 0.214 | 0.557 |
| Connectedness (Con) | 0.916 | 0.951 | 1 |
| Efficiency | 0.95 | 0.877 | 0.62 |
| Hierarchy | 0 | 0 | 0 |
| Lubness | 1 | 1 | 1 |

**Table S16 Network nodes’ centrality indices of microbial communities of water, shrimp and sediment habitats in the SCPE at six culture stages**

See the excel file.
