# Supplementary figures and images for: Environmental Water and Sediment Microbial Communities Shape Intestine Microbiota for Host Health: The Central Dogma in an Anthropogenic Aquaculture Ecosystem
Source: Front Microbiol. 2021 Nov 2;12:772149. doi: 10.3389/fmicb.2021.772149 (PMC8593368; doi:10.3389/fmicb.2021.772149)

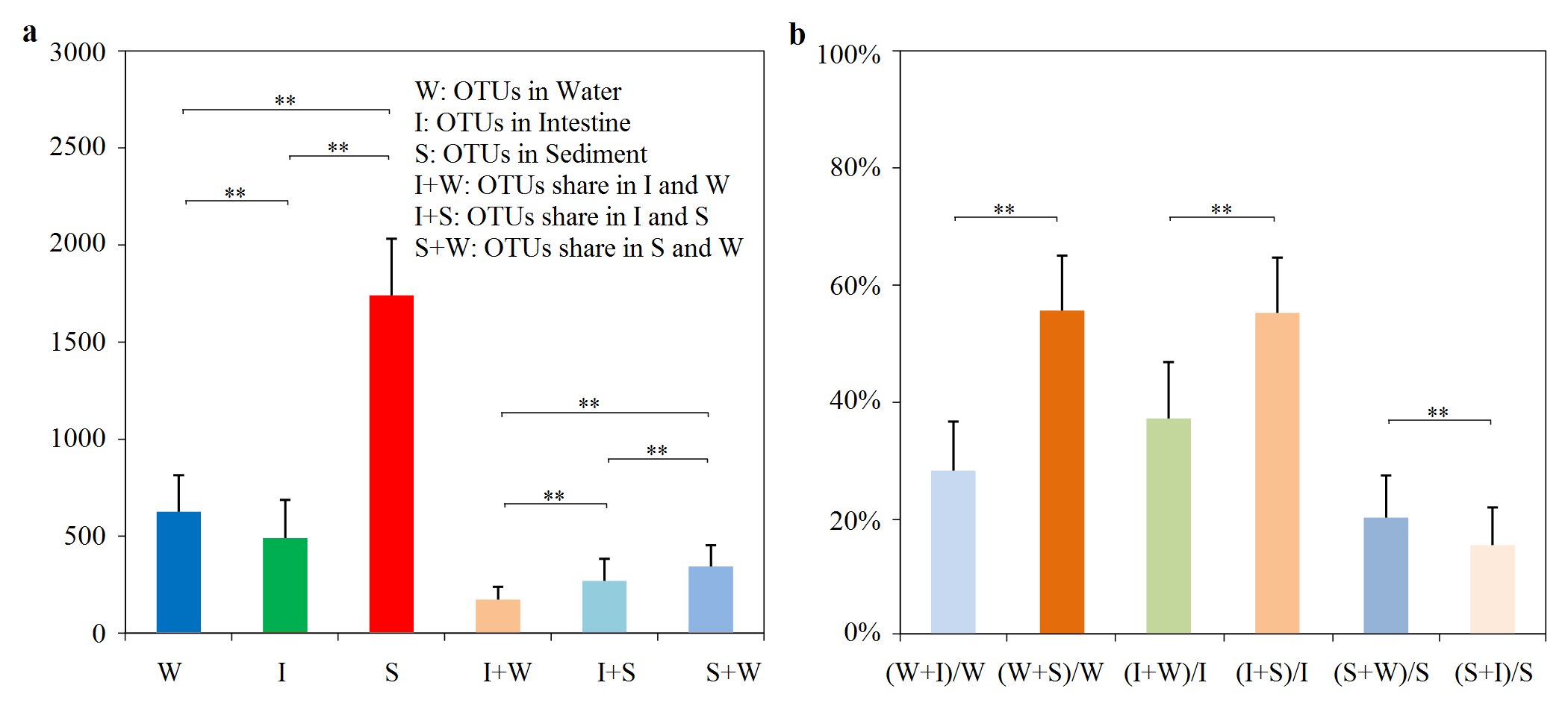

Supplement: Supplementary file 1 [file Data_Sheet_1.ZIP › Supplementary Materials/Supplementary Figures/FIGURE S1.tif]

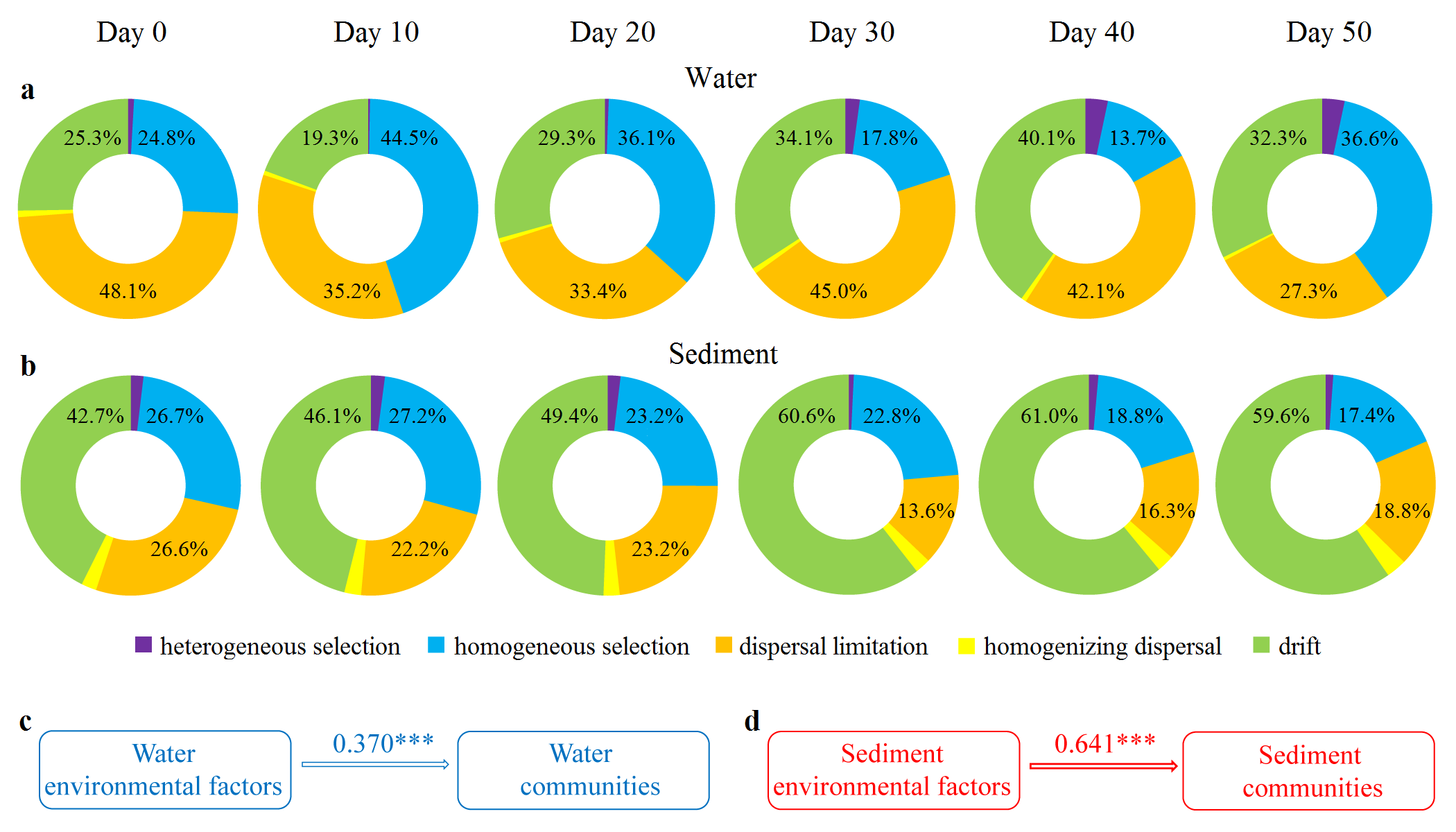

Supplement: Supplementary file 1 [file Data_Sheet_1.ZIP › Supplementary Materials/Supplementary Figures/FIGURE S10.tif]

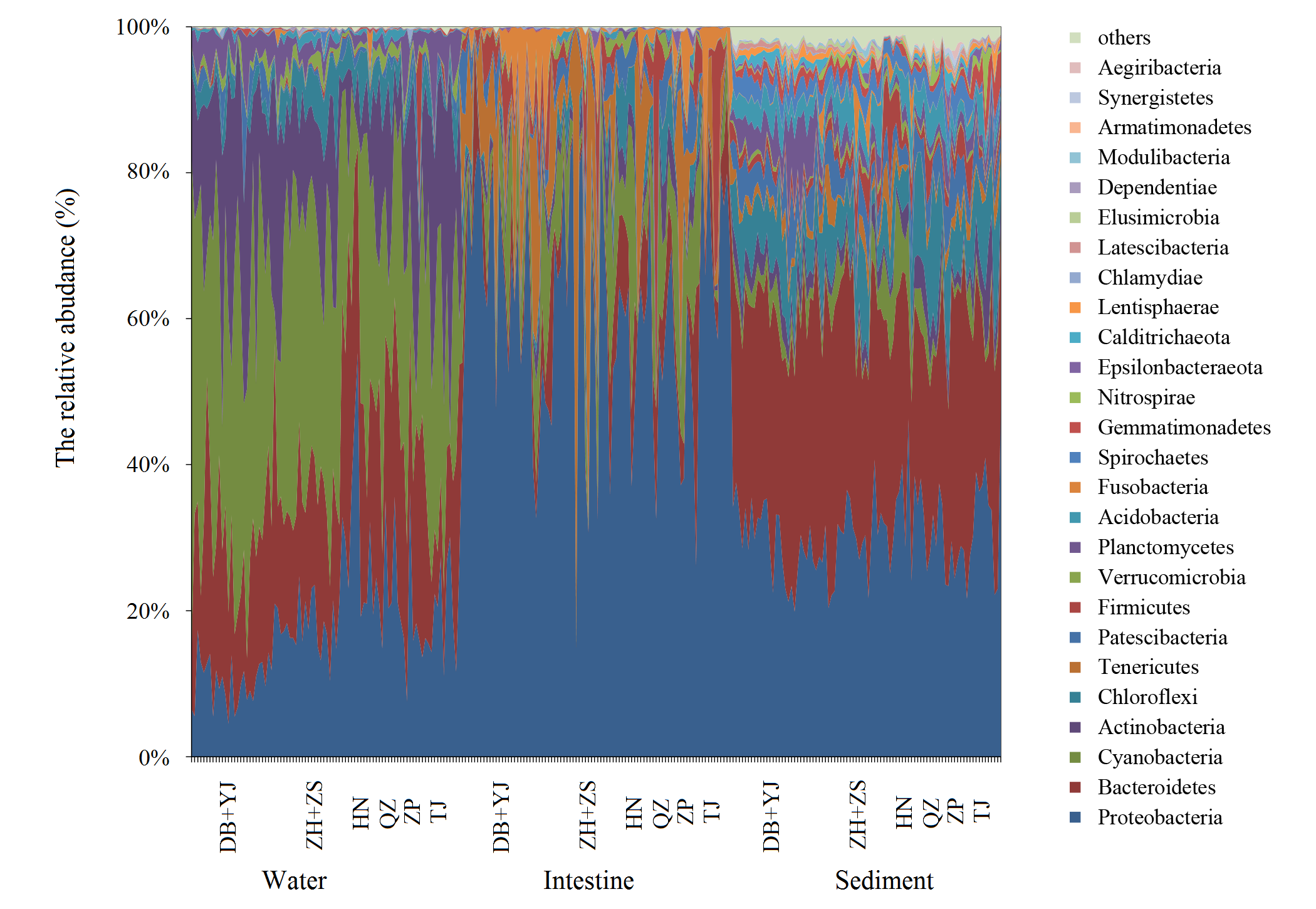

Supplement: Supplementary file 1 [file Data_Sheet_1.ZIP › Supplementary Materials/Supplementary Figures/FIGURE S2.tif]

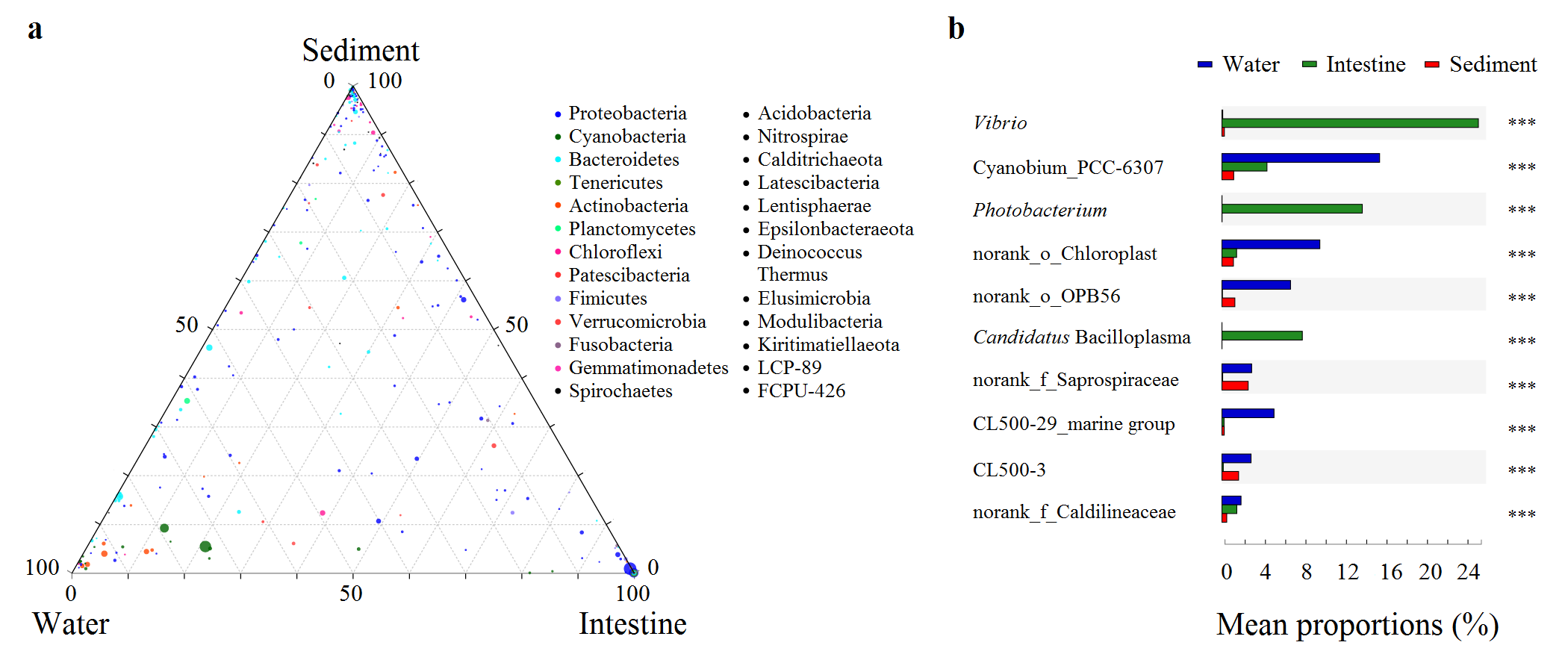

Supplement: Supplementary file 1 [file Data_Sheet_1.ZIP › Supplementary Materials/Supplementary Figures/FIGURE S3.tif]

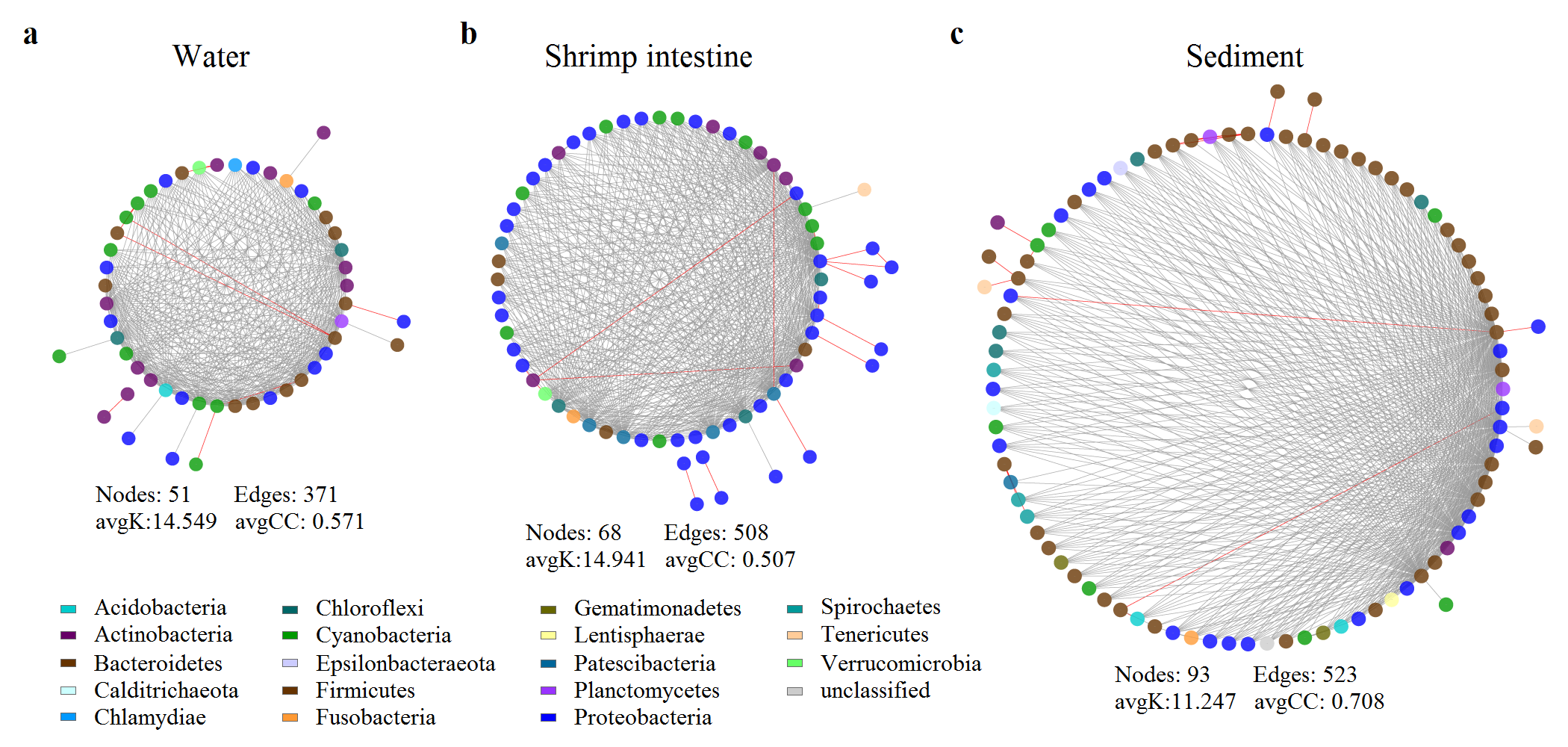

Supplement: Supplementary file 1 [file Data_Sheet_1.ZIP › Supplementary Materials/Supplementary Figures/FIGURE S4.tif]

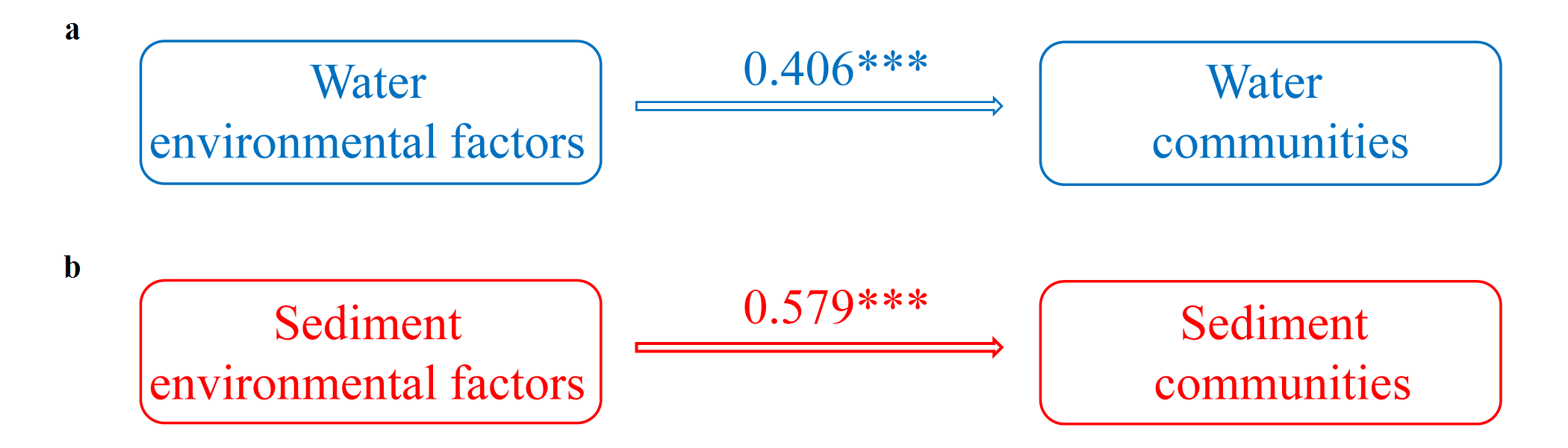

Supplement: Supplementary file 1 [file Data_Sheet_1.ZIP › Supplementary Materials/Supplementary Figures/FIGURE S5.tif]

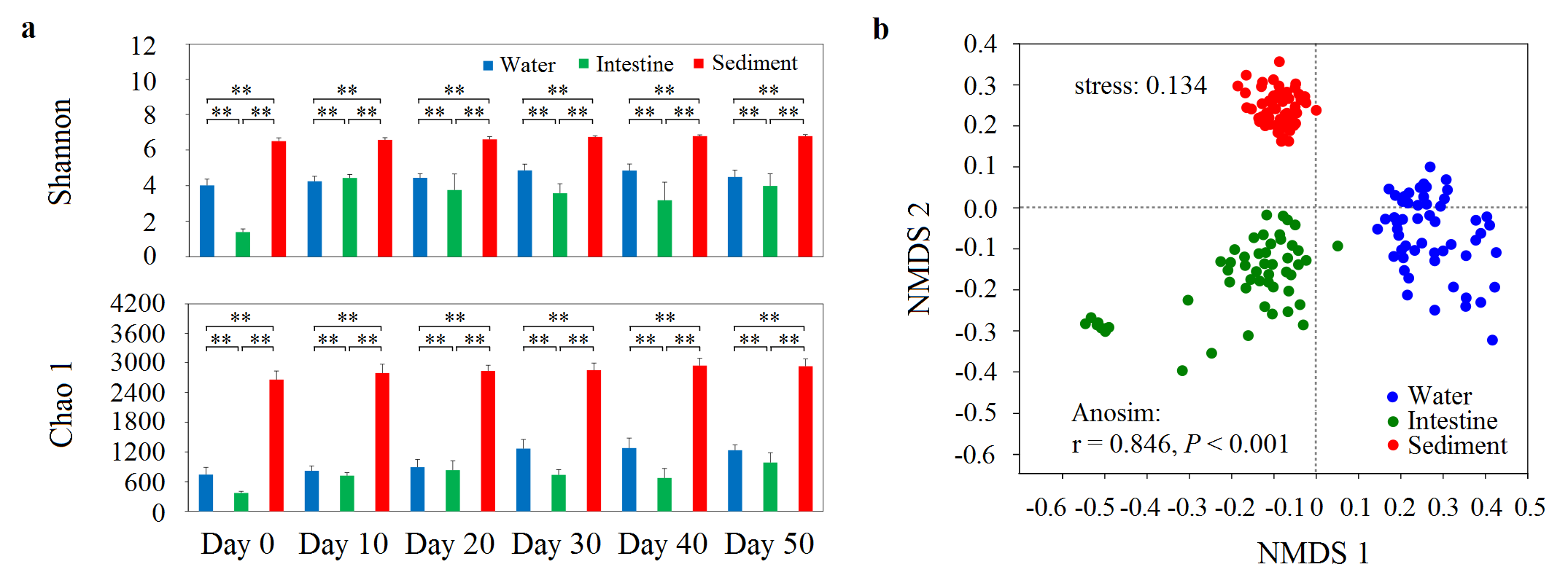

Supplement: Supplementary file 1 [file Data_Sheet_1.ZIP › Supplementary Materials/Supplementary Figures/FIGURE S6.tif]

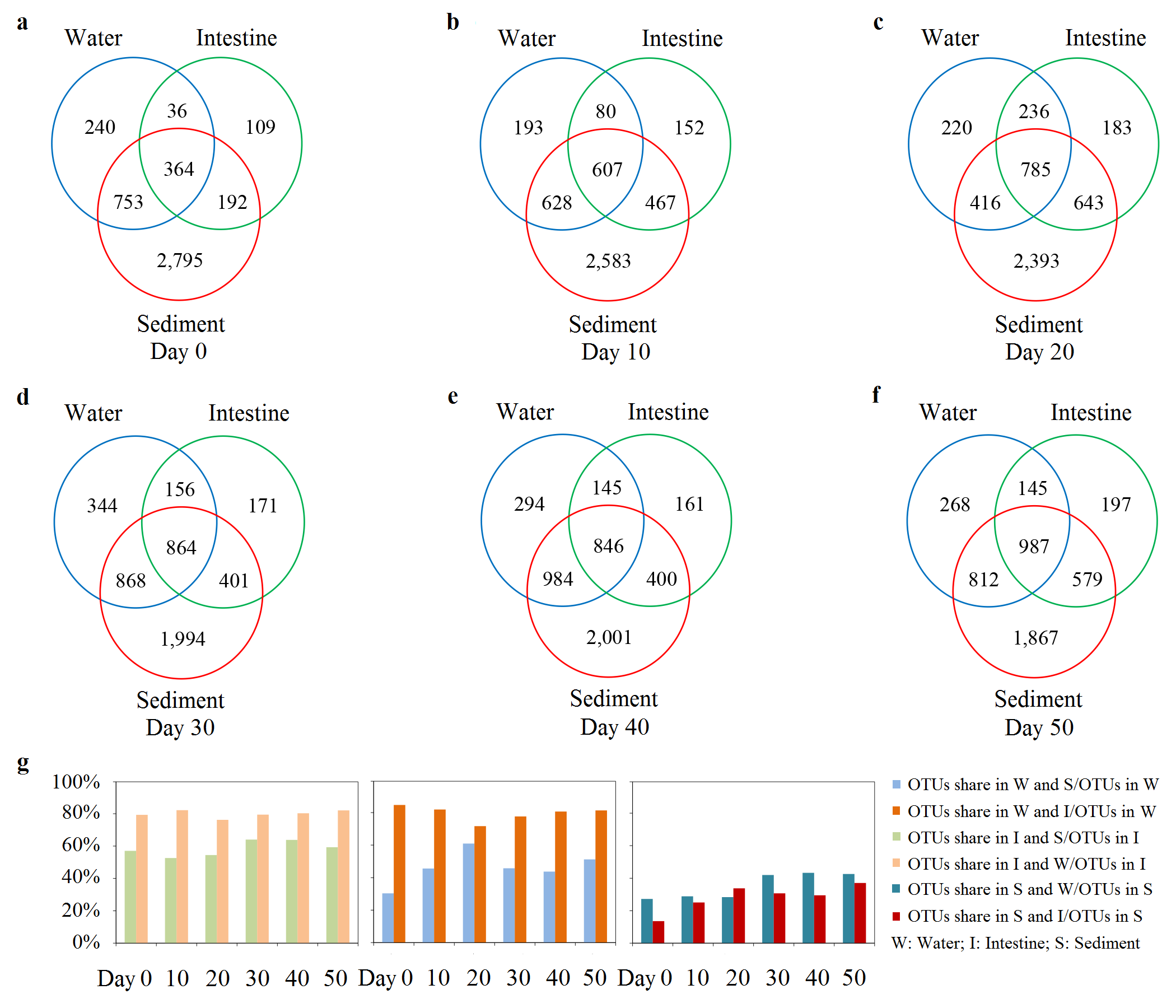

Supplement: Supplementary file 1 [file Data_Sheet_1.ZIP › Supplementary Materials/Supplementary Figures/FIGURE S7.tif]

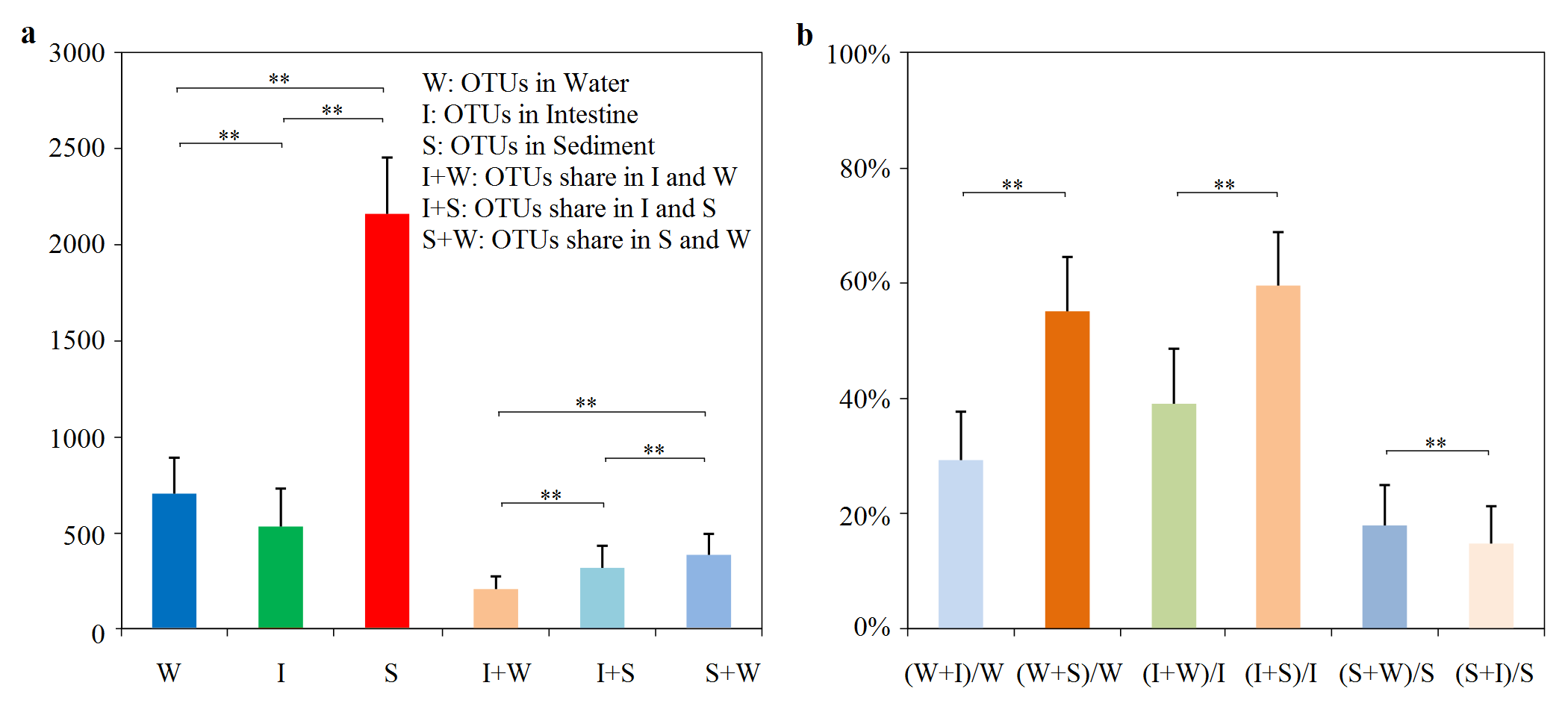

Supplement: Supplementary file 1 [file Data_Sheet_1.ZIP › Supplementary Materials/Supplementary Figures/FIGURE S8.tif]

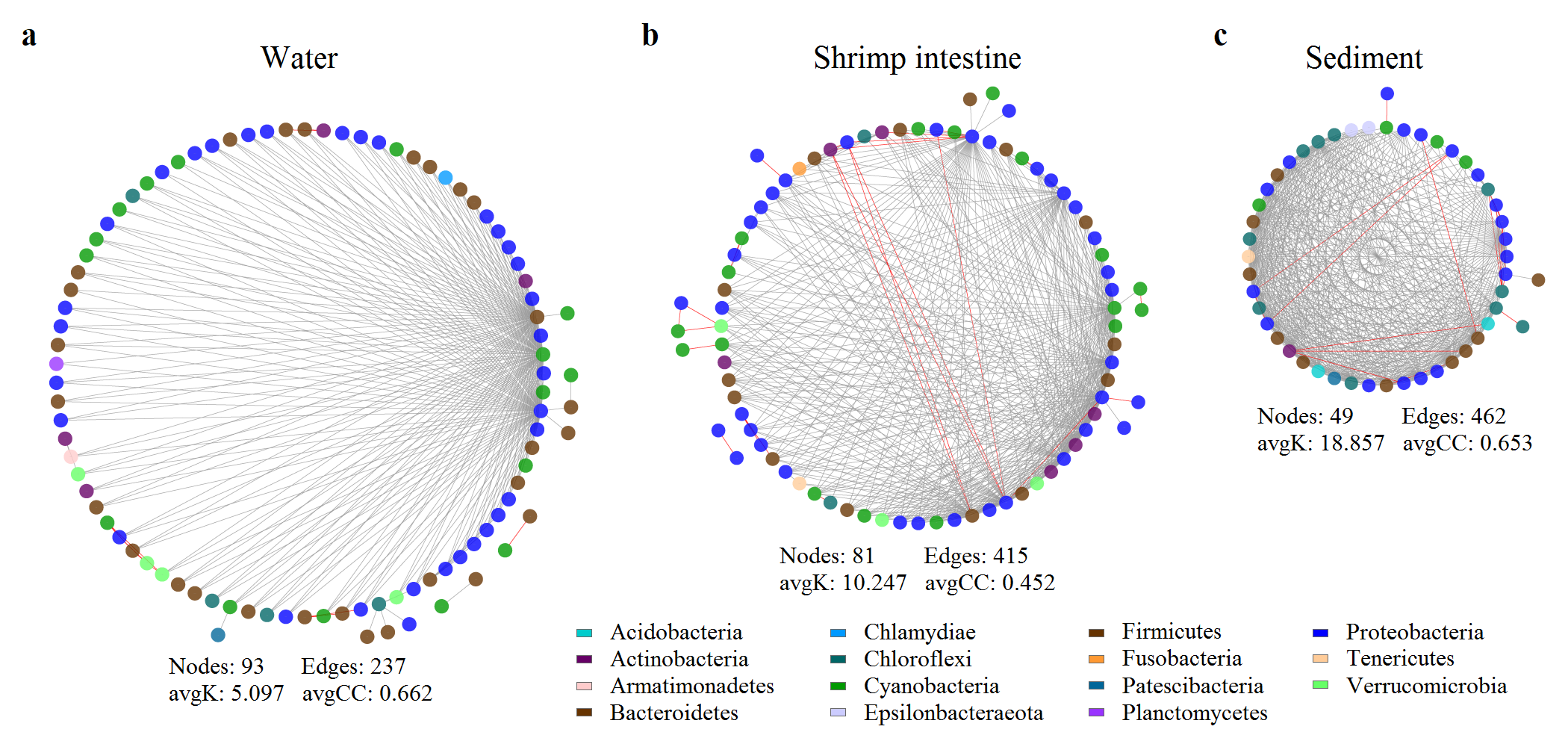

Supplement: Supplementary file 1 [file Data_Sheet_1.ZIP › Supplementary Materials/Supplementary Figures/FIGURE S9.tif]
